# Green fluorescent genetically encoded calcium indicator based on calmodulin/M13-peptide from fungi

**DOI:** 10.1371/journal.pone.0183757

**Published:** 2017-08-24

**Authors:** Natalia V. Barykina, Oksana M. Subach, Kiryl D. Piatkevich, Erica E. Jung, Aleksey Y. Malyshev, Ivan V. Smirnov, Andrey O. Bogorodskiy, Valentin I. Borshchevskiy, Anna M. Varizhuk, Galina E. Pozmogova, Edward S. Boyden, Konstantin V. Anokhin, Grigori N. Enikolopov, Fedor V. Subach

**Affiliations:** 1 Moscow Institute of Physics and Technology, Dolgoprudny, Moscow Region, Russia; 2 P.K. Anokhin Institute of Normal Physiology of RAMS, Moscow, Russia; 3 National Research Center “Kurchatov Institute”, Moscow, Russia; 4 MIT Media Lab, Massachusetts Institute of Technology, Cambridge, MA, United States of America; 5 Institute of Higher Nervous Activity and Neurophysiology of RAS, Moscow, Russia; 6 Medico-Biological Faculty, N.I. Pirogov Russian National Research Medical University, Moscow, Russia; 7 Federal Research and Clinical Center of Physical-Chemical Medicine of Federal Medical Biological Agency, Moscow, Russia; 8 Engelhardt Institute of Molecular Biology RAS, Moscow, Russia; 9 MIT McGovern Institute for Brain Research, MIT, Cambridge, MA, United States of America; 10 Lomonosov Moscow State University, Moscow, Russia; 11 Department of Anesthesiology, Stony Brook University Medical Center, Stony Brook, NY, United States of America; 12 Center for Developmental Genetics, Stony Brook University, Stony Brook, NY, United States of America; Russian Academy of Medical Sciences, RUSSIAN FEDERATION

## Abstract

Currently available genetically encoded calcium indicators (GECIs) utilize calmodulins (CaMs) or troponin C from metazoa such as mammals, birds, and teleosts, as calcium-binding domains. The amino acid sequences of the metazoan calcium-binding domains are highly conserved, which may limit the range of the GECI key parameters and cause undesired interactions with the intracellular environment in mammalian cells. Here we have used fungi, evolutionary distinct organisms, to derive CaM and its binding partner domains and design new GECI with improved properties. We applied iterative rounds of molecular evolution to develop FGCaMP, a novel green calcium indicator. It includes the circularly permuted version of the enhanced green fluorescent protein (EGFP) sandwiched between the fungal CaM and a fragment of CaM-dependent kinase. FGCaMP is an excitation-ratiometric indicator that has a positive and an inverted fluorescence response to calcium ions when excited at 488 and 405 nm, respectively. Compared with the GCaMP6s indicator *in vitro*, FGCaMP has a similar brightness at 488 nm excitation, 7-fold higher brightness at 405 nm excitation, and 1.3-fold faster calcium ion dissociation kinetics. Using site-directed mutagenesis, we generated variants of FGCaMP with improved binding affinity to calcium ions and increased the magnitude of FGCaMP fluorescence response to low calcium ion concentrations. Using FGCaMP, we have successfully visualized calcium transients in cultured mammalian cells. In contrast to the limited mobility of GCaMP6s and G-GECO1.2 indicators, FGCaMP exhibits practically 100% molecular mobility at physiological concentrations of calcium ion in mammalian cells, as determined by photobleaching experiments with fluorescence recovery. We have successfully monitored the calcium dynamics during spontaneous activity of neuronal cultures using FGCaMP and utilized whole-cell patch clamp recordings to further characterize its behavior in neurons. Finally, we used FGCaMP *in vivo* to perform structural and functional imaging of zebrafish using wide-field, confocal, and light-sheet microscopy.

## Introduction

Calcium ions are universal second messengers involved in the regulation of physiological responses in a wide range of organisms. Genetically encoded calcium indicators (GECIs) are indispensable tools for *in vivo* visualization of calcium dynamics. GECIs are composed of a sensory Ca^2+^-binding domain (e.g., calmodulin or troponin C) that, when bound to Ca^2+^, causes a conformational change that induces a fluorescence readout from the attached fluorescent proteins (FPs). GECI can be divided into two classes based on the number of FPs they contain. In single-FP-based GECIs, the change in FP domain fluorescence is modulated by changes in the sensory module induced by binding of Ca^2+^ ions [1,2]. Typically, the sensory module is located on the N- or C-terminus of the GECI and includes mammalian calmodulin (CaM) that undergoes Ca^2+^-dependent binding with the M13 peptide of myosin light chain kinase (CaM/M13) fused to the opposite end of the indicator. However, these features vary slightly among the many types of GECI that currently exist. For example, two current calcium indicators, Camgaroo 1 and 2, do not contain the M13 peptide, and the mammalian CaM is inserted inside enhanced yellow FP (EYFP) [3,4]. Another recently developed calcium indicator, NTnC, contains a truncated troponin C (TnC) from toadfish inserted into the mNeonGreen green FP [5]. Apart from single-FP-based GECIs, double-FP-based GECIs take advantage of the physical property of fluorescent molecules called fluorescence resonance energy transfer (FRET). FRET involves one FPs emitting photons that then gets absorbed as well as excites an adjacent FP, if in close enough proximity, which then emits a photon of the wavelength of the adjacent FP [6]. The Ca^2+^-binding domain of FRET-based sensors employs a mammalian CaM/M13 pair [7] or a mammalian or toadfish TnC [8]. The GECIs described and current publicationsindicate a potential issue, that the diversity of domains in the sensory modules are limited to proteins from metazoa such as mammals, birds, and teleosts.

Low diversity is an issue because the key properties of GECI, such as Ca^2+^-binding affinity, and association/dissociation kinetics, as well as interactions with other cellular components, are defined by the sensory module. Indeed, modifications of CaM and/or M13 peptide have been used to alter the affinity to Ca^2+^, extending the range from 15 nM of Ca^2+^ for YC-Nano15 [7] to 2 mM for Ca-G1' [9]. The kinetics of Ca^2+^ dissociation for GECIs also varies widely, ranging from 0.33 s^-1^ for YC-Nano140 [7] up to 62 s^-1^ for GCaMP3_fast_ [10]. The first attempts to develop GECIs revealed that GECIs based on a mammalian CaM/M13 pair exhibited poor functional properties when expressed in the brain of transgenic animals. Compared to the purified protein, Cameleon FRET sensors were ten-fold less responsive to calcium when expressed in the central nervous system of transgenic mice [11]. Fluorescence recovery after photobleaching (FRAP) experiments revealed that 50% of the Inverse-pericam calcium indicator was immobile both in the cell body and the axons of transgenic mice neurons [12]. Regardless of calcium concentration approximately 15% of mammalian CaM in the brain is bound to membranes. The dramatic reductions in the dynamic range of GECIs and their reduced mobility may be explained by interactions of the mammalian CaM/M13 pair with other cellular components, such as CaM, kinases, phosphatases, ion channels and others [13]. To overcome these limitations, mutations were introduced into both CaM and the M13 peptide [14,15] or differently-sourced Ca^2+^-binding proteins, such as TnC, were used [8]. However, the commonly used versions of green calcium GECIs such as G-GECOs and GCaMP6s were not modified to prevent interactions with mammalian CaMs.

Because the sensory modules of GECIs define their key characteristics and since the diversity of sensory modules in the available GECIs is limited to metazoa, we decided to explore the proteins from another kingdom, fungi, as a source of sensory modules with novel properties and reduced interactions with endogenous mammalian proteins. All CaMs from metazoa share high amino acid sequence homology, whereas CaMs from fungi and yeasts have lower sequence homology to their metazoan orthologues. Therefore, we decided to use CaMs, M13-like peptides, or calcineurins from the fungi *Aspergillus niger* and *Aspergillus fumigatus* and the yeast *Komagataella pastoris*, since their sequences differ greatly from those in metazoa, i.e. selected domains shared only 66–85, 25–47 and 59–68% amino acid identity with their metazoan counterparts, respectively. Here we have designed a single-FP-based green ratiometric GECI, designated FGCaMP, that contains CaM and the M13-like peptide from *Aspergillus* fungi and circularly-permutated EGFP. We validated its spectral, physicochemical and kinetics characteristics *in vitro* using standard techniques including stopped-flow fluorimetry. We found point mutations within the M13-like peptide and CaM that enhanced the affinity and fluorescence response of FGCaMP to calcium ions. Once developed, we were able to use FGCaMP to monitor Ca^2+^ changes in mammalian cells and in neurons during voltage-induced and spontaneous activities. Additionally, we characterized the properties of FGCaMP in neurons using whole-cell patch clamp recording. Finally, we performed structural and functional imaging of FGCaMP *in vivo* in zebrafish.

## Results and discussion

### Development of calcium indicators based on Ca^2+^-binding proteins from fungi

**Fig 1:**
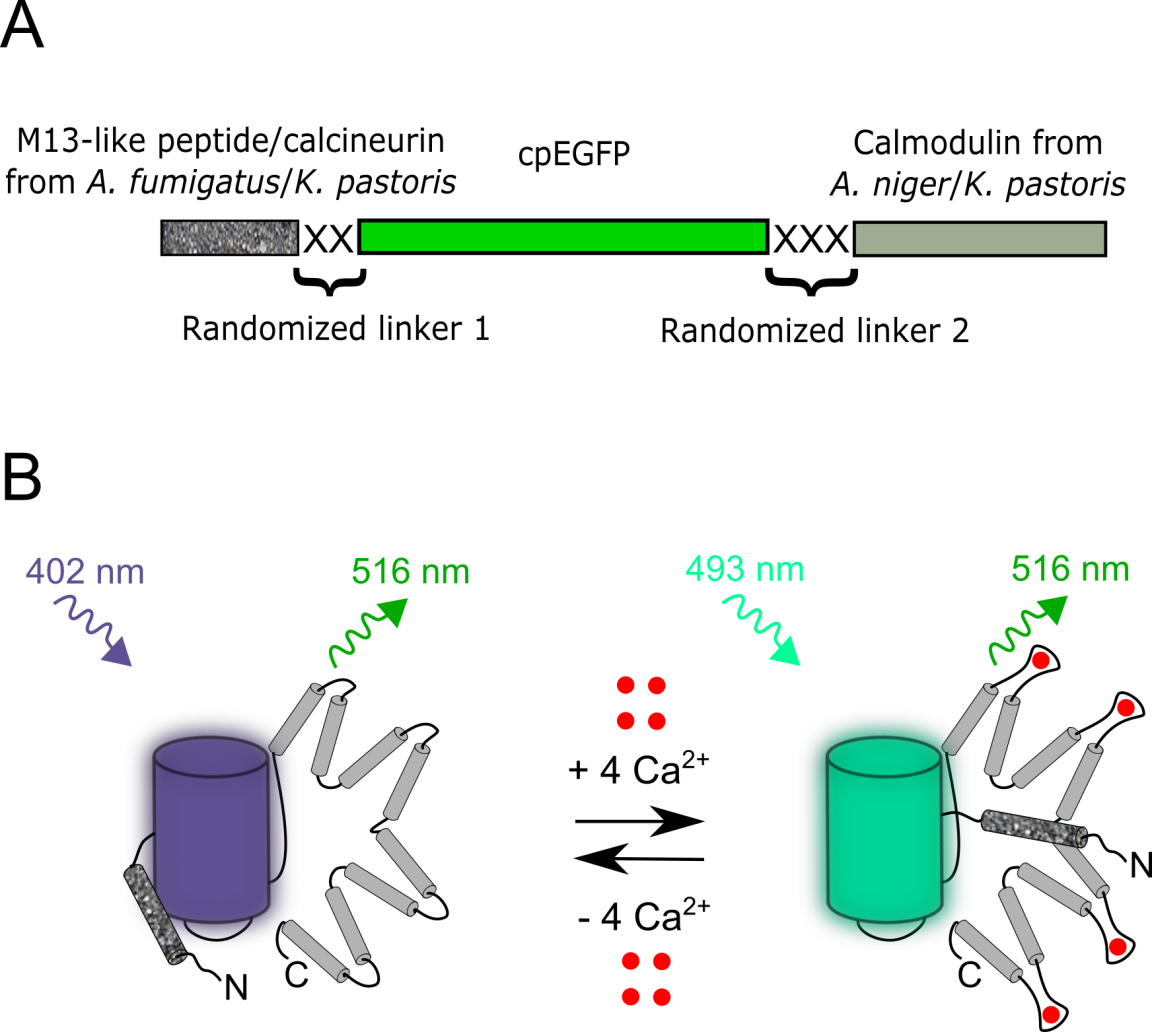
Schematic representation of the composition of the original libraries and suggested stages of the FGCaMP calcium indicator function. (A) The designed original libraries for calcium indicators consisted of M13-like peptide or calcineurin from *A*. *fumigatus or K*. *pastoris* and CaM from *A*. *niger* or *K*. *pastoris* as a sensory part and cpEGFP as a fluorescent part, with randomized linkers located between sensory and fluorescent components. (B) Schematic representation of the FGCaMP indicator function. The cpEGFP component is shown as blue and cyan cylinders according to the excitation wavelengths; CaM and M13-like peptide are shown in light gray and speckled gray, respectively; Ca^2+^ ions are shown as red dots.

To engineer GECIs with sensory parts from fungi, we cloned CaMs from fungi and assembled gene libraries with randomized linkers between fluorescent and sensory domains. We aligned amino acids sequences of CaMs, M13-like peptides and calcineurins from *Aspergillus niger*, *Aspergillus fumigatus* fungi and *Komagataella pastoris* yeast, which are available from the Genomic database (Figure A in S1 Supporting Information). These sequences were homologous but different from the mammalian CaMs (66–85% amino acids identity, ident), M13-like peptides (25–47% ident) and calcineurins (59–68% ident) which are present in available GECIs. The cloned CaMs from *Aspergillus niger* fungus and *Komagataella pastoris* yeast were further used to assemble four bacterial libraries composed of CaMs, cpEGFP from GCaMP6f variant [16] and M13-like peptides or calcineurins (Fig 1A). Both the linkers between sensory domains and cpEGFP were randomized and had the length of two and three amino acids.

Next, the generated libraries of sensors with randomized linkers were expressed in *E*. *coli* periplasm and screened for the response to Ca^2+^ ions as described previously [5]. Briefly, we screened ~10,000 colonies per library for the largest fluorescence response to Ca^2+^ ions on Petri dishes. Analysis of the best variants on bacterial suspensions revealed that the variants having pairs of [CaM from *K*. *pastoris*/M13 peptide from *K*. *pastoris*], [CaM from *K*. *pastoris*/calcineurin from *K*. *pastoris*], [CaM from *A*. *niger*/calcineurin from *A*. *fumigatus*] and [CaM from *A*. *niger*/M13 peptide from *A*. *fumigatus*] demonstrated positive contrasts for Ca^2+^ addition of 0, 15, 23 and 68%, respectively. Thus, the variant pair demonstrating the highest dynamic range, CaM from *A*. *niger* and M13 peptide from *A*. *fumigatus* fungi, was chosen as a template for further improvement.

The mutant with the largest response for Ca^2+^ from the library with CaM from *A*. *niger* and M13 peptide from *A*. *fumigatus* fungi were subjected to the two rounds of random mutagenesis and selection. The final mutant was named FGCaMP (F stands for fungi) and had 10 mutations relative to the original template including the linkers (Figure B in S1 Supporting Information). Three mutations were located in the fluorescent domain and were external to the β-barrel, and two mutations resided in the sensory part. None of the mutations in the sensory domain were located within Ca^2+^-binding loops (EF-hands) that are responsible for Ca^2+^ binding.

### *In vitro* characterization of the FGCaMP calcium indicator

**Fig 2:**
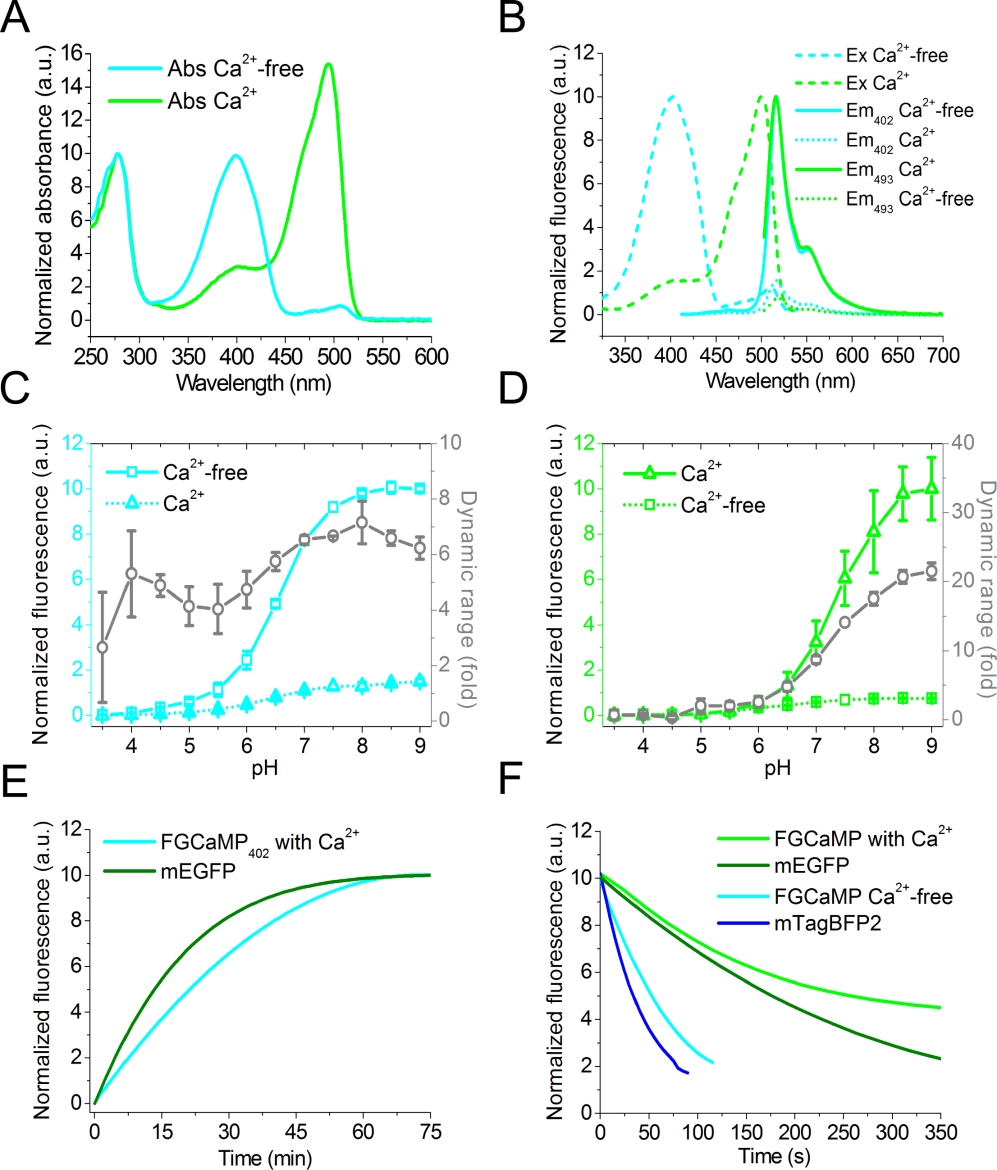
*In vitro* properties of the purified FGCaMP indicator. (A, B) Absorbance (A), excitation and emission spectra (B) for FGCaMP in Ca^2+^-free and Ca^2+^-bound states. (C, D) Intensity and dynamic range for FGCaMP as a function of pH at 402 (C) and 490 nm excitation (D), respectively. The dynamic range (fold) at each pH value was measured as the ratio of FGCaMP fluorescence intensity in the absence of Ca^2+^ to that in the presence of Ca^2+^ at 402 nm excitation (C) and vice versa at 490 nm excitation (D). Error represents the standard deviation for the average of three records. (E) Maturation curves for mEGFP and FGCaMP in Ca^2+^-bound state at 402 nm excitation. (F) Photobleaching curves for FGCaMP in Ca^2+^-free state (at 355 nm excitation), in Ca^2+^-bound state (at 470 nm excitation), mEGFP, and mTagBFP2.

The key properties of the FGCaMP calcium indicator were further characterized *in vitro* on the protein purified from bacteria in comparison with GCaMP6s GECI. In the Ca^2+^-free and Ca^2+^-saturated states, FGCaMP has absorbance/fluorescence maxima at 402/516 and 493/516 nm, respectively (Fig 2A and 2B and Table 1). The 402 nm and 493 nm absorbing forms of FGCaMP can be attributed to the protonated and anionic forms of GFP chromophore, respectively. Upon addition of 39 μM free Ca^2+^, FGCaMP demonstrated 6.9-fold decrease and 14.7-fold increase in fluorescence when exited at 402 and 493 nm, respectively. Hence, FGCaMP is a ratiometric GECI with maximal 101-fold fluorescence ratio change upon Ca^2+^ binding. The brightness of the 402- and 493-forms of FGCaMP in its Ca^2+^-free and Ca^2+^-saturated states is 7-fold higher or similar to that for respective states of control GCaMP6s (Table 1). The “ratiometric-pericam” and GEX-GECO1 ratiometric green GECIs have substantially lower brightness and fluorescence ratio changes of 10- and 18-fold, respectively [17,18] (Table A in S1 Supporting Information). The ratiometric yellow GECI, Y-GECO1, has significant 200-fold fluorescence ratio change, but it suffers from the low brightness of its 413 nm absorbing form, which is 13-fold dimmer than the 402 nm absorbing form of FGCaMP [19]. In addition, Y-GECO1 has inverted response to Ca^2+^ ions as compared to FGCaMP.

**Table 1:** *In vitro* characterization of FGCaMP indicator.

Properties		Proteins			
		FGCaMP		GCaMP6s	
		apo	sat	apo	sat
**Absorbance maxima (nm)**		402	493	402	500
**Emission maxima (nm)**		516		518	515
**Quantum yield** ^**a**^		0.48±0.02	0.46±0.01	0.11±0.01	0.61
**ε (mM**^**-1**^**cm**^**-1**^**)** ^**b**^		55±5	106±20	33.3±0.6	77±3
**Brightness (%)**		56	104	7.8	100
**Fluorescence contrast (fold)**	**402-form**	6.9±0.5		ND	
	**493-form**	14.7±0.6		44±6	
**pKa**_**402**_ ^**c**^		6.56±0.03 [>10]	7.0±0.6 [7.52±0.01]	ND	
**pKa**_**493**_ ^**c**^		6.2±0.2 [7.1±0.3]	7.33±0.07 [7.34±0.08]	9.6±0.3	6.16±0.08
**K**_**d**_ **(nM)** ^**d**^	**402-form**	276±9 [n = 2.8±0.3]		ND	
	**493-form**	273±7 [n_1_ = 3.5±0.3]; 4700±200 [n_2_ = 1.9±0.2]		144±9 [n = 2.5±0.3]	
**K**_**d**_ **(nM) with 1 mM MgCl**_**2**_ ^**d**^	**402-form**	400±60 [n = 2.3±0.6]		ND	
	**493-form**	460±40 [n_1_ = 2.8±0.4] 4400±800 [n_2_ = 1.9±0.2]		192±7 [n = 2.4±0.2]	
**K**_**d**_^**kin**^ **(nM)** ^**d**^	**402-form**	200±60 [n = 3.32±0.05]		ND	
	**493-form**	200±60[n = 3.33±0.05]		160±40 [n = 3.22±0.05]	
**k**_**on**_ **(s**^**-1**^**× M**^**-n**^**)** ^**e**^	**402-form**	(2.1±0.1) × 10^22^		ND	
	**493-form**	(2.5±0.1) × 10^22^		(6.5±0.9) × 10^21^	
**t**_**1/2**_^**off**^ **(s)** ^**f**^	**402-form**	0.65±0.01		ND	
	**493-form**	0.66±0.01		0.88±0.01	
**k**^**onset**^_**limit**_ **(s**^**-1**^**)** ^**g**^	**402-form**	39±5		ND	
	**493-form**	43±5		300±20	
**Protein state**		monomer		monomer	
**Maturation half-time (min)** ^**h**^		ND	27±4	ND	
**Photobleaching half-time (sec)** ^**i**^		54±9	260±40	110±20	190±20

^a^ GCaMP6s in the Ca^2+^-saturated state (QY  =  0.61) and mTagBFP2 (QY  =  0.64) were used as reference standards for 493- and 402-nm absorbing states, respectively.

^b^ Extinction coefficient was determined by alkaline denaturation.

^c^ pKa values were determined according to pH dependence of fluorescence. pKa values determined according to the absorbance dependence vs pH (Figure C in S1 Supporting Information) are shown in square brackets.

^d^ Experimental data for 402- and 493-forms were fitted to Hill equation or y = V_1_*x^n1^/(K_d1_^n1^+x^n1^) + V_2_*x^n2^/(K_d2_^n2^+x^n2^), respectively. Hill coefficient is shown in square brackets.

^e^ Hill coefficients and k_on_ values were obtained via fitting the observed association rates (Figure F in S1 Supporting Information) at 0–350 nM Ca^2+^ concentrations to the equation k_obs_  =  k_on_ × [Ca^2+^]^n^ + k (Fig 3C). K_d_^kinetic^  =  (k_off_/k_on_)^1/n^. k_off_ values were determined from the dissociation kinetics curves (Fig 3D). Hill coefficients are shown in square brackets.

^f^ t_1/2_^off^ values were determined from the dissociation kinetics curves (Fig 3D).

^g^ k^onset^_limit_ values are saturation levels of the observed association rates (at >600–800 nM Ca^2+^ concentrations; Figure G in S1 Supporting Information).

^h^ mEGFP had a maturation half-time of 14 min; we could not estimate the maturation rate for GCaMP6s because of its low expression level in bacteria. Maturation of FGCaMP was registered according to 402-form in buffer B containing 10 mM Ca^2+^.

^i^ mEGFP had a photobleaching half-time of 170 ± 20 sec; mTagBFP2 had a photobleaching half-time of 32 ± 6 sec.

We next characterized pH stability of the FGCaMP indicator. According to fluorescence changes in the Ca^2+^-free and Ca^2+^-saturated states, the 402 nm absorbing form of FGCaMP had slightly different fluorescence pKa values of 6.56 and 7.0, respectively (Fig 2C and 2D and Table 1). Upon Ca^2+^ binding, the apparent fluorescence pKa value of 493 nm absorbing form of FGCaMP showed a larger shift from 6.2 to 7.33. Notably, in contrast to most previously reported GECOs and GCaMPs variants [16,18], in which the apparent fluorescence pKa values shifted toward a lower value upon binding to Ca^2+^, pKa of FGCaMP shifted toward larger value similar to the Y-GECO1 ratiometric sensor [19]. However, pKa values for FGCaMP in the Ca^2+^-free and Ca^2+^-saturated states determined according to absorbance at 402 or 493 nm (Figure C in S1 Supporting Information and Table 1) were different from respective pKas calculated according to fluorescence; the exception was similar fluorescence and absorbance pKa values for 493 nm absorbing form in the Ca^2+^-saturated state. According to absorbance spectra chromophore of FGCaMP_apo_ in Ca^2+^-free state is mostly in protonated state in the range of all pH tested (pKa > 10). The difference between fluorescence and absorbance pKa values could reflect the changes in quantum yield of FGCaMP with a variation of pH.

We next characterized the efficiency of maturation of the purified FGCaMP indicator. At 37°C, according to fluorescence of 402 nm absorbing form, in the presence of Ca^2+^ FGCaMP matured 1.9-fold slower than mEGFP (Fig 2E and Table 1). Because of poor expression levels of GCaMP6s, we could not fully characterize its maturation rate.

We further characterized the photostability of the purified FGCaMP protein. Under a wide-field microscope equipped with a metal halide lamp and 470/40BP excitation filter, FGCaMP sensor in the Ca^2+^-saturated state photobleached 1.5-fold slower than mEGFP (Fig 2F). In the absence of Ca^2+^ FGCaMP photobleached 1.7-fold slower than a control mTagBFP2 when illuminated using 355/25BP excitation light. Hence, the FGCaMP indicator exceeded control proteins in terms of photostability.

Both in the absence of Ca^2+^ and in the presence of 1 mM Ca^2+^, purified FGCaMP sensor eluted on size-exclusion chromatography as monomer (Figure D in S1 Supporting Information); note that monomeric proteins are usually preferable in terms of reduced cytotoxicity [20].

**Fig 3:**
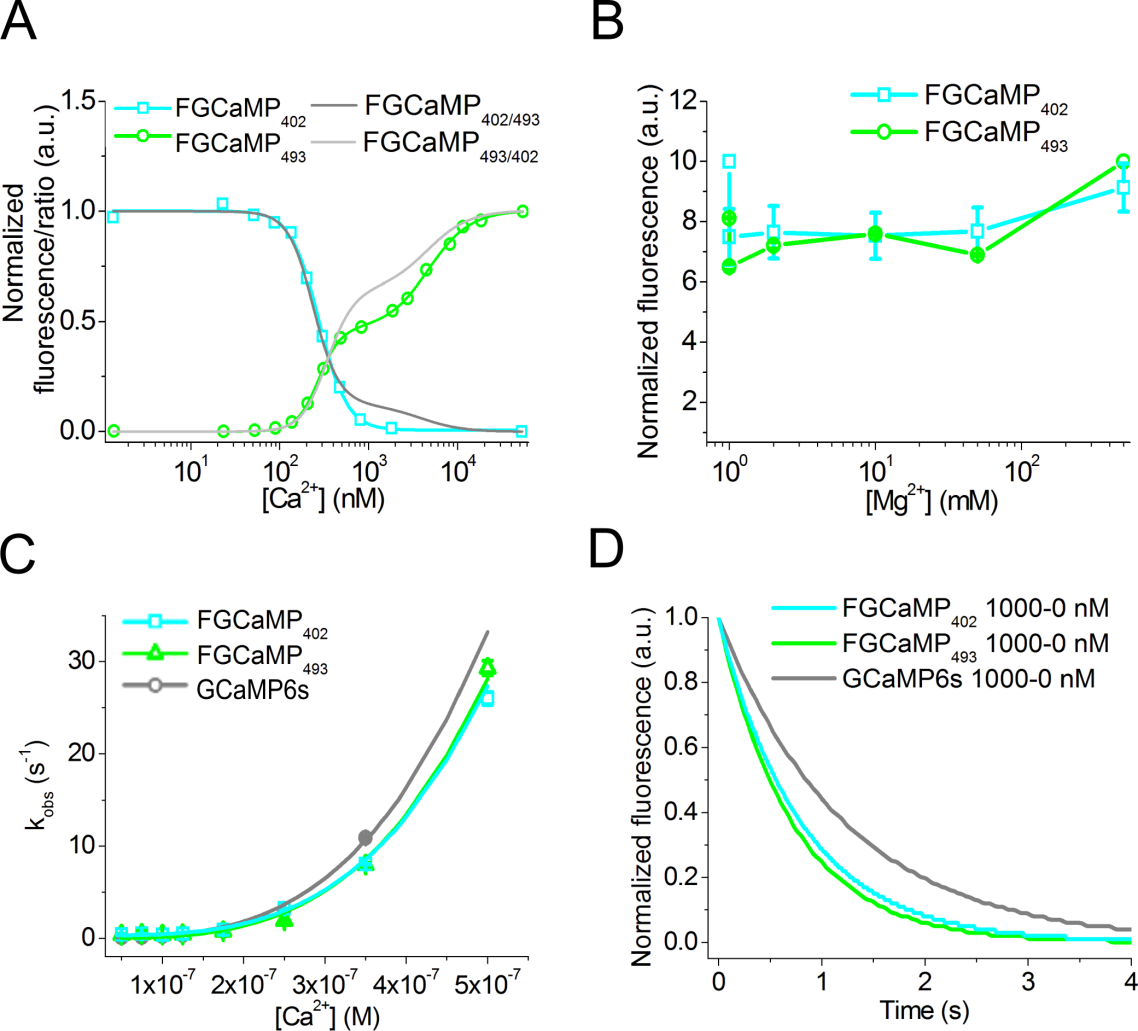
K_d_ and Ca^2+^-binding kinetics of FGCaMP. (A) Ca^2+^ titration curves for 402- and 493-forms of FGCaMP using 402 and 490 nm excitation light, respectively. Fluorescence changes were normalized to maximal and minimal values. Experimental data for 402- and 493-forms were fitted to Hill equation or double Hill equation y = V_1_*x^n1^/(K_d1_^n1^+x^n1^) + V_2_*x^n2^/(K_d2_^n2^+x^n2^), respectively. The value of 1 was added to normalized fluorescence changes for both forms and their ratio was calculated. (B) Magnesium titration curves for FGCaMP sensor. (C) Association kinetics. Observed Ca^2+^ association rate constants determined from stopped-flow experiments at low Ca^2+^ concentrations (in the range of 0–350 nM for GCaMP6s and 0–500 nM for FGCaMP) are overlaid with the fitted curves (k_obs_ = k_on_ x [Ca^2+^]^n^ + k_off_). (D) Dissociation kinetics. Fluorescence changes were normalized to maximal and minimal values. Starting concentration was 1000 nM.

To determine the affinity of FGCaMP to Ca^2+^ ions we performed equilibrium binding experiments. According to the equilibrium binding titration curves, FGCaMP displayed a biphasic Ca^2+^ binding with two K_d_ dissociation constants of 273 nM and 4.7 μM when excited at 493 nm (Table 1 and Fig 3A). Fluorescence response corresponding to the high-affinity binding component of the FGCaMP titration curve (“contrast K_d1_”) was 5.5-fold higher than that for the low-affinity binding component (“contrast K_d2_”) (Table B in S1 Supporting Information). According to Hills coefficients, both high- and low-affinity binding sites bound Ca^2+^ with high cooperativity. When excited at 402 nm, FGCaMP demonstrated monophasic Ca^2+^ binding with K_d_ of 276 nM value that was similar to that for the high affinity binding component of the 493 nm absorbing form. Hence, at the low Ca^2+^ concentrations around 300 nM, FGCaMP behaves as a ratiometric sensor. At Ca^2+^ concentrations significantly higher or lower than 300 nM one of the 493 or 402 nm absorbing forms is not fluorescent and FGCaMP can be considered as intensiometric GECI. The mammalian CaM/M13 hybrid protein without GFP and CaM/M13-based sensor Cameleon-1 demonstrated similar biphasic Ca^2+^ binding with two dissociation constants of 70–80 nM and 2–11 μM [6,21]. The equilibrium K_d_ value for the high-affinity binding site of the FGCaMP indicator was 1.9-fold higher than the K_d_ value for GCaMP6s but 1.4-fold smaller than the K_d_ value for the GCaMP6f sensor whose cpEGFP part was used for designing FGCaMP [16]. Because the K_d_ values for both GCaMP6s and GCaMP6f sensors are optimized for the detection of calcium neuronal activity, we assumed that FGCaMP with the similar high affinity K_d_ value should be also appropriate for the detection of neuronal activity.

To characterize the specificity of FGCaMP to Ca^2+^ ions we performed titration of FGCaMP in the presence of magnesium ions (Mg^2+^) that are present at ~0.5–11 mM concentration in the cytoplasm of neurons [22]. Equilibrium binding titration of FGCaMP with Mg^2+^ showed that the FGCaMP fluorescence did not noticeably change up to a 50 mM concentration of Mg^2+^ (Fig 3B). In the presence of 1 mM Mg^2+^, purified FGCaMP and GCaMP6s demonstrated slight increases of high-affinity K_d_ values in 1.4–1.7- and 1.3-fold, respectively (Table 1 and Figure E in S1 Supporting Information). The addition of 1 mM Mg^2+^ ions practically did not affect K_d_ value for the lower affinity site of FGCaMP. Hence, FGCaMP has a high specificity to Ca^2+^ ions.

### Characterization of the FGCaMP calcium indicator using stopped-flow fluorimetry

Ca^2+^ association and dissociation kinetics for FGCaMP were further studied using stopped-flow fluorimetry in comparison with GCaMP6s GECI. To investigate association kinetics we calculated the observed association rate constants (k_obs_) by fitting the kinetic curves (Figure F in S1 Supporting Information) to the double exponentials (Figure G in S1 Supporting Information). At low Ca^2+^ concentrations of 0–350/500 nM the curves could also be fitted to single exponentials (k_obs_ = k1, Figure G in S1 Supporting Information) since the contributions of the second exponential were insignificant (Figure G in S1 Supporting Information, panel B). k_obs_ values (k1 and k2) increased nonlinearly with increasing Ca^2+^ concentrations indicating binding stoichiometry different from 1:1. k_obs_ values reached saturation (k^onset^_limit_) at concentrations higher than 600 nM (in the case of GCaMP6s) or 800 nM (in the case of FGCaMP). FGCaMP was 7-fold slower than GCaMP6s in terms of the onset limiting rate (k^onset^_limit_ (FGCaMP) = 43±5 s^-1^, k^onset^_limit_ (GCaMP6s) = 300±20 s^-1^; Figure G in S1 Supporting Information, panel A). Hence, at high (> 600 nM) Ca^2+^ concentrations FGCaMP binds Ca^2+^ significantly slower than does GCaMP6s.

Fitting of the observed association rate constants at low (in the range of 0–350/500 nM) Ca^2+^ concentrations to the equation k_obs_ = k_on_ × [Ca^2+^]^n^ + k provided actual association rate constants (k_on_), and Hill coefficients (Fig 3C and Table 1). k_on_ value for FGCaMP was 3.8-fold higher than that for GCaMP6s. Hence, at low (< 600 nM) Ca^2+^ concentrations FGCaMP binds Ca^2+^ faster than does GCaMP6s. According to the Hill coefficient values, FGCaMP and GCaMP6s bind Ca^2+^ with similar cooperativity. K_d_^kin^ values for FGCaMP and GCaMP6s indicators were rather similar to those determined from the equilibrium studies. Calculations based on the data obtained for FGCaMP using excitation at 500 or 402 nm, gave almost similar results. We were unable to investigate thoroughly binding kinetics for the low-affinity binding site of FGCaMP, because the responses were too fast for accurate measurements at Ca^2+^ concentrations higher than 800 nM. The high affinity binding site of FGCaMP probably associates with Ca^2+^ ions with a lower rate than does low-affinity site.

Next, we studied Ca^2+^ dissociation kinetics for FGCaMP. To study dissociation from the high-affinity site of the FGCaMP indicator alone we used initial Ca^2+^ concentrations of 1000 or 350 nM at which the high-affinity site is occupied, and the low-affinity site is mostly unoccupied (Fig 3A). The dissociation half-times (t_1/2_^off^) and k_off_ rate constants were obtained from the dissociation kinetic curves (Fig 3D). The t_1/2_^off^ of 0.65±0.01 s and 0.66±0.01 s coincided for both 402- and 493-forms of FGCaMP, respectively. As compared with FGCaMP, t_1/2_^off^ for GCaMP6s was 1.3-fold longer (t_1/2_^off^ = 0.88±0.01 s). Hence, at low Ca^2+^ concentrations, FGCaMP releases Ca^2+^ ions slightly faster than does GCaMP6s and this correlates with slightly higher K_d_ value for high-affinity site of FGCaMP as compared with that for GCaMP6s.

### Improving FGCaMP properties by site-directed mutagenesis in sensory part

We next attempted to enhance the fluorescence response to low Ca^2+^ concentrations and affinity of the FGCaMP indicator to Ca^2+^ ions using site-directed mutagenesis of the sensory part. First, we introduced known mutations in CaM part at positions that enhanced properties of other GCaMP-like indicators such as GCaMP3 (N60D), GCaMP6s (D78Y/T79R), and GCaMP8 (M36L) [16,23]. These positions were located within Ca^2+^-binding loops (EF hand 2) of CaM or out of them (Figure H in S1 Supporting Information). We also probed mutations Q26T, N98D, and D138N located in EF hands 1, 3 and 4, respectively that are present in NTnC or GCaMP6s indicators. The estimated Ca^2+^ affinities determined for the respective 402- and 493-forms for all mutants were similar. Probably, the protonated 402-form directly transforms into anionic 493-form. FGCaMP/N60D/D78Y/T79R/N98D mutant called FGCaMP2 combined both low K_d1_ value for high-affinity binding site and 1.4-fold reduced “contrast K_d2_” at high Ca^2+^ concentrations (Figure I in S1 Supporting Information and Table B in S1 Supporting Information). According to the data for FGCaMP/N60D, FGCaMP/N98D and FGCaMP/D78Y/T79R mutants, N98D single mutation was responsible for the low K_d1_ value and D78Y/T79R double mutation reduced “contrast K_d2_” value at high Ca^2+^ concentrations. Other mutations and their combinations increased K_d1_ values in 1.2–2.9-fold and changed K_d2_ values maximally in 1.3–1.5-fold. Most mutations and their combinations practically did not affect contrasts for 402-form and “contrast K_d1_” values. Finally, we selected FGCaMP2 beneficial mutant for further directed mutagenesis.

We next introduced mutations Q26D, T28D, T62D, F100D and S102D in FGCaMP2 in order to further increase the affinity of FGCaMP to Ca^2+^ ions as a result of decreasing K_d1_ and/or K_d2_ values. These mutations located in EF hands 1, 2 and 3 and were negatively charged to enhance binding to positively charged Ca^2+^ ions. Only mutation S102D decreased high-affinity K_d1_ value in 1.4-fold, but all other mutations increased K_d1_ value in 1.5–3.2-fold (Figure J in S1 Supporting Information and Table B in S1 Supporting Information). Tested mutations increased low-affinity K_d2_ values in 1.3–2.9-fold except T62D mutation which decreased K_d2_ value in 2.3-fold. Fluorescence contrasts for 402-form slightly varied within 1.1–1.4-fold, but “contrast K_d1_” and “contrast K_d2_” values for 493-form were practically the same. The FGCaMP3 (FGCaMP/N60D/D78Y/T79R/N98D/S102D) mutant having lowest high-affinity K_d1_ value was chosen for further mutagenesis.

Next, we examined the influence of mutations in the M13-like peptide of FGCaMP3 on its Ca^2+^ binding affinity. With this aim, we generated two bacterial libraries of FGCaMP3 with randomized non-conserved residues located in tripeptide …TLH… or …IDT… (Figure H in S1 Supporting Information). From these libraries we selected mutants having bright fluorescence and high contrast. Further analysis of these mutants revealed that mutagenesis at M13-like peptide had a pronounced effect on FGCaMP3 binding affinity to Ca^2+^ ions (Figure K in S1 Supporting Information and Table B in S1 Supporting Information). High-affinity K_d1_ values for FGCaMP3 mutants with T3L/H5K, I8V/D9N/T10I or I8V/D9C/T10V mutations were in 2.0–5.3-fold lower than that for FGCaMP3. Mutations I8R/D9G/T10A and T3M/L4N/H5L substantially increased K_d1_ value in 3.8- and 5.0-fold, respectively. All found mutations affected K_d2_ value to a less extent and reduced it in 1.2–2.0-fold. Mutations in M13-like peptide also affected fluorescence contrasts of 402-form in 1.3–1.5-fold, decreased “contrast K_d1_” values for 493-form in 1.4–2.4-fold and practically did not change “contrast K_d2_” values except mutation T3M/L4N/H5L where it was increased in 1.7-fold. Therefore, the affinity of FGCaMP indicator to Ca^2+^ ions can be adjusted by the mutations in the M13-like peptide.

Hence, we have found mutants of FGCaMP with enhanced affinity and fluorescence response to low Ca^2+^ concentrations which may be used as templates for the development of improved versions of FGCaMP in the future.

### Imaging of the FGCaMP indicator in HeLa Kyoto mammalian cells

**Fig 4:**
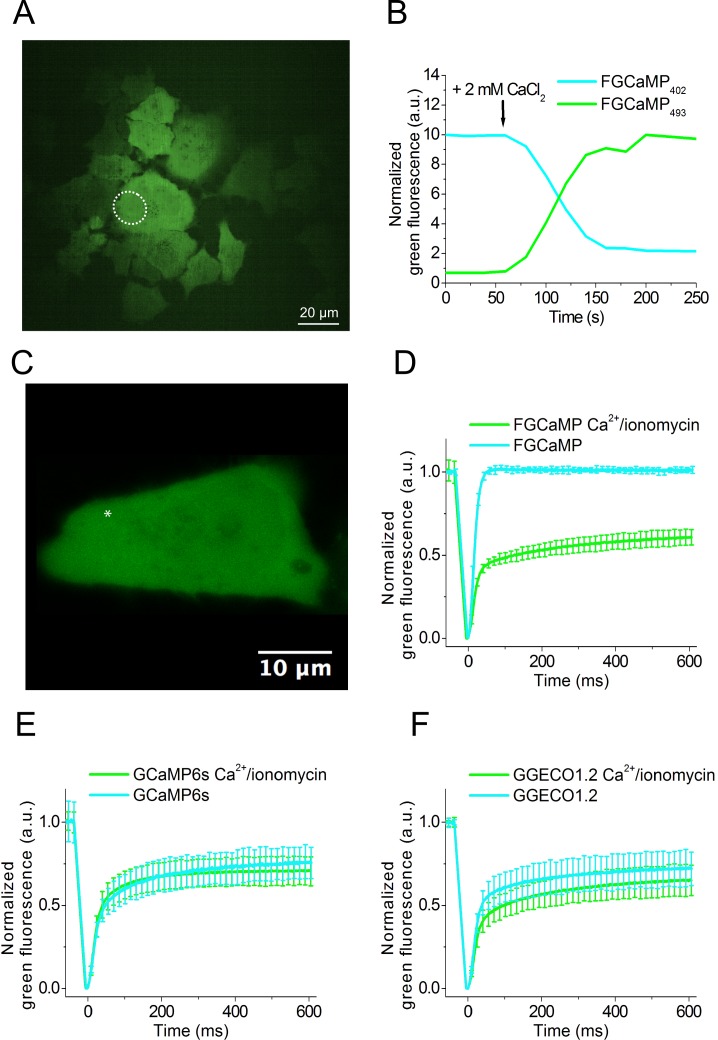
Response of FGCaMP to Ca^2+^ concentration changes in HeLa Kyoto cells and FRAP of FGCaMP and control GECIs at different Ca^2+^ concentrations in HeLa Kyoto cells. (A) Confocal image of HeLa Kyoto cells expressing FGCaMP calcium sensor. (B) The graph illustrates changes in the green fluorescence of FGCaMP in HeLa Kyoto cells excited at 405 (cyan line) or 488 nm (green line) in response to the addition of 2 mM CaCl_2_ and 5 μM ionomycin. The changes correspond to the area indicated with white circles on the panel A. (C) Example of HeLa Kyoto cells expressing FGCaMP calcium sensor used for FRAP experiment. An example of FRAP area having a size of around 1 μm^2^ is indicated with a white asterisk. (D)-(F) The graphs illustrate FRAP induced changes in green fluorescence of FGCaMP and control GECIs at physiological Ca^2+^ concentrations and in response to the addition of 2 mM CaCl_2_ and 5 μM ionomycin. Error bars are standard deviations shown for each 20^th^ dot on plots.

To validate the behavior of FGCaMP in live mammalian cells, it was expressed in the cytoplasm of HeLa Kyoto mammalian cells. First, we characterized the dynamic range of the FGCaMP sensor. With this aim, we recorded the response of FGCaMP to changes in cytoplasmic Ca^2+^ concentration according to previously described protocol [18]. After addition of external ionomycin/CaCl_2,_ we observed 4-fold drop and 14-fold increase in green fluorescence when excited at 405 nm and 488 nm, respectively (Fig 4A and 4B).

To assess possible interactions of FGCaMP protein with the intracellular environment we performed fluorescence recovery after photobleaching (FRAP) of FGCaMP experiments in the cytoplasm of HeLa cells. With this aim, FRAP experiments were carried out in HeLa Kyoto cells transiently transfected with FGCaMP and control GCaMP6s and G-GECO1.2 GECIs at physiological and elevated Ca^2+^ concentrations (Fig 4C–4F). At physiological Ca^2+^ concentrations in the case of FGCaMP fluorescence recovered to 100% after photobleaching (Fig 4D). At the same conditions fluorescence of control GCaMP6s and G-GECO1.2 recovered to only 70–74%. (Fig 4E and 4F). This result evidences that about 30% of GCaMP6s and G-GECO1.2 were not freely diffusible, and they might be bound to calmodulin or other cellular proteins, but FGCaMP was freely diffusible in the cytoplasm of HeLa cells at physiological Ca^2+^ concentrations. In the presence of ionomycin/2 mM Ca^2+^ all three sensors behaved similarly, i.e. 60–70% of them were mobile. For FGCaMP this change in mobility was reversible (Figure L in S1 Supporting Information). Hence, at elevated Ca^2+^ concentrations, part of FGCaMP became not freely diffusible. Earlier R. Tsien and co-workers inserted several mutations in M13 peptide and CaM parts of the Cameleons GECIs in order to disrupt interactions with an excess of cellular CaM [15]. These mutations led to an increase of the dynamic range and sensitivity of Cameleons in neuronal culture. However, these mutations are absent in GCaMP6s and G-GECO1.2 and this may be the reason why they have limited mobility inside mammalian cells. Therefore, the origin of CaM and M13-peptide parts of the FGCaMP indicator from fungi led to a calcium indicator that does not interfere with, nor is perturbed by, endogenous cellular proteins inside mammalian cells at physiological Ca^2+^ concentrations.

### Imaging spontaneous Ca^2+^ activity in dissociated neuronal culture expressing FGCaMP

**Fig 5:**
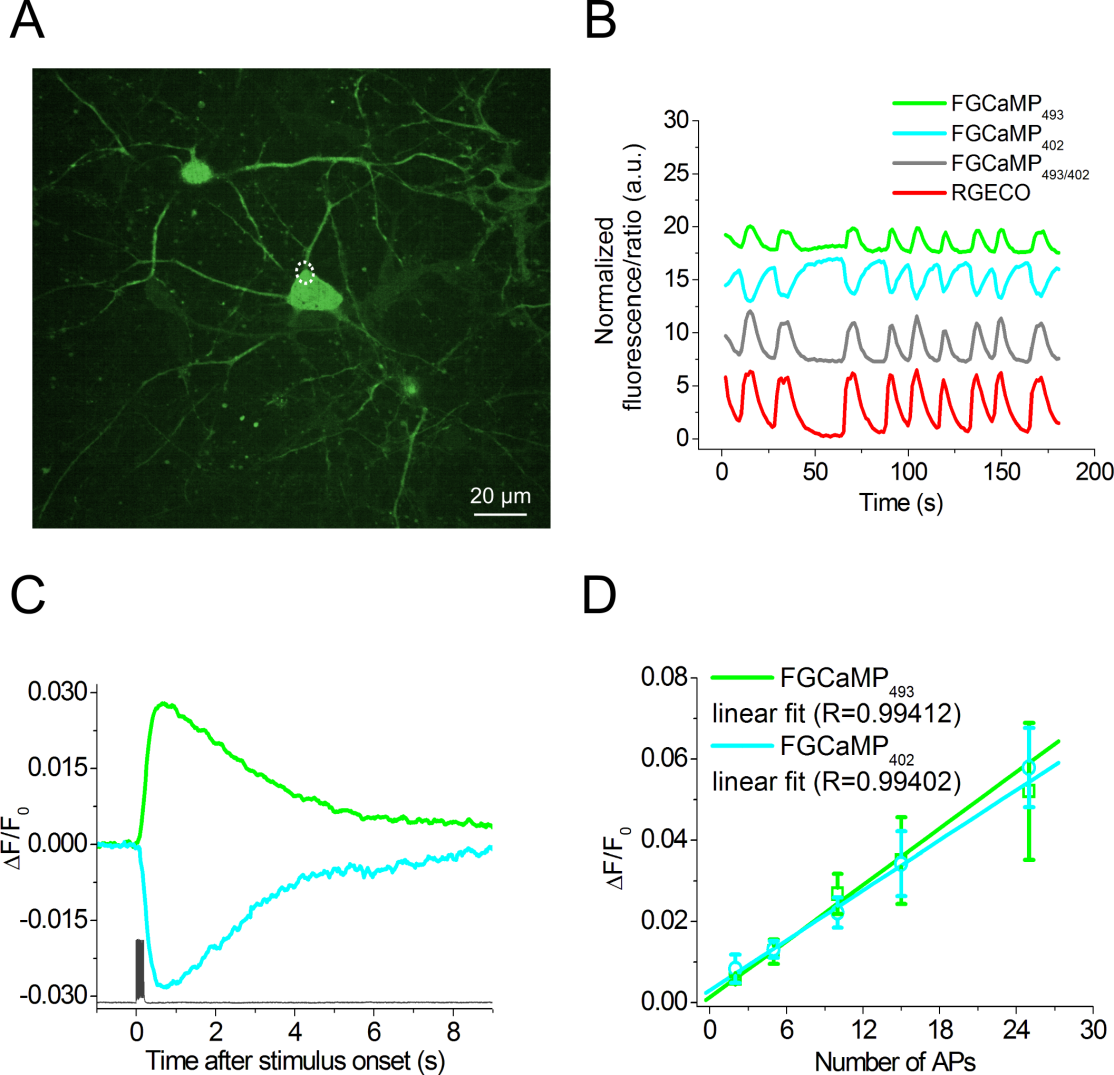
Response of FGCaMP to Ca^2+^ concentration variations as a result of spontaneous activity and to intracellularly induced APs in cultured neurons. (A) Dissociated neuronal culture co-expressing FGCaMP and R-GECO calcium sensors. Red channel for R-GECO is not shown. (B) The graph illustrates changes in red fluorescence of R-GECO (red line, excitation 561 nm) and green fluorescence of FGCaMP for 402- (cyan line, excitation 405 nm) or 493-form (green line, excitation 488 nm) as a result of spontaneous activity in neuronal culture. The gray line represents the ratio between fluorescence intensities for FGCaMP in two channels with excitation at 488 and 405 nm, respectively. The graphs illustrate changes in fluorescence in the area indicated with a white circle. (C) Fluorescence changes in FGCaMP-expressing cells induced by the train of 10 APs (bottom) for 402- (cyan curve, excitation 400 nm) or 493-form (green line, excitation 470 nm). Ca^2+^ responses were averaged across all recorded neurons in different wells. An example of intracellular recording (dark gray) was taken from one cell. (D) Dependence of the amplitudes of responses induced by different numbers of APs in neurons expressing FGCaMP indicator recorded for 493- (green line, N = 7, excitation 470 nm) and 402-forms (cyan line, N = 7, excitation 400 nm). Note that in the range of 2 to 25 APs, the dependence is linear for both excitation wavelengths. Values are shown as the means ± SEM.

We further demonstrated FGCaMP capability of detecting spontaneous activity in dissociated neuronal culture using confocal microscopy and compared it with R-GECO1. We transduced neuronal cultures isolated from mice at age of P0-P2 with recombinant adeno-associated viral particles (rAAVs) carrying FGCaMP or control R-GECO1 indicators under CAG promoter. Using FGCaMP we successfully visualized spontaneous cytosolic Ca^2+^ oscillations in 2–3 weeks old neuronal cultures according to the fluorescence of both 402- and 493-forms (Fig 5B). The rise half-times for FGCaMP and R-GECO1 expressing in the same neurons were the same, i.e. 2.5±1.1 and 1.9±0.2 sec, respectively. The decay half-times for FGCaMP and R-GECO1 were also similar, i.e. 3.7±0.9 and 4.0±0.8 sec, respectively. FGCaMP demonstrated 16% decrease and 14% increase in fluorescence when excited at 405 and 488 nm, respectively. ΔR/R ratiometric signal of 31% for FGCaMP was slightly less than ΔF/F intensiometric signal of 44% for the control R-GECO1. Hence, FGCaMP is applicable for monitoring of spontaneous neuronal activity.

### Characterization of the FGCaMP sensor in a dissociated neuronal culture with a whole-cell patch clamp and an external electric field

To further characterize the performance of FGCaMP in neurons, we compared fluorescence responses to intracellular stimulation of cultured neurons, expressing FGCaMP and GCaMP6s using whole-cell patch recording. In one series of these experiments, we measured the fluorescence changes in neurons in response to the train of 10 action potentials (APs) induced intracellularly at a 50 Hz frequency. Cultured neurons were transduced with rAAV vectors carrying the gene of the appropriate sensor 7–10 days prior to the experiment. In cells expressing FGCaMP responses to APs were collected with 400 or 470 nm excitation successively. Altogether 7 cells in 4 wells with FGCaMP and 6 cells in 2 wells with GCaMP6s were recorded. Both indicators demonstrated fast and reliable changes in fluorescence levels in response to a train of 10 APs. As expected, intracellular stimulation of GCaMP6s-expressing neurons induced an increase in green fluorescence, while in response to the same stimulation FGCaMP-expressing cells showed a drop or increase in green fluorescence using 400 or 470 nm excitation light, respectively (Fig 5C). Neurons expressing FGCaMP showed faster kinetics of the Ca^2+^ responses as compared with GCaMP6s-positive cells. Thus both half-rise and half-decay times were significantly shorter for 402-form of FGCaMP (p<0.005, Student’s t-test) (Table 2). At the same time, the mean ΔF/F values for the train of 10 APs for FGCaMP- and GCaMP6s-expressing neurons were 0.03 and 0.5, respectively. The signal-to-noise ratio (SNR) and ΔF/F were significantly in 11–16- and 17-fold greater for Ca^2+^ responses measured in GCaMP6s-expressing neurons in comparison to FGCaMP-expressing cells recorded with both 400 and 470 nm excitation light (p<0.01, Student’s t-test).

**Table 2:** Characteristics of FGCaMP and GCaMP6s calcium indicators responses to electrical intracellular stimulation of neurons expressing these indicators.

Protein	Number of cells	Number of APs	Rise half-time, s ^a^	Decay half-time, s ^b^	ΔF/ F ^c^	SNR ^d^
FGCaMP (493-form)	7	10	0.28±0.01	2.1±0.4	0.029±0.006	17±5
FGCaMP (402-form)	7	10	0.29±0.02	2.0±0.2	0.030±0.007	12±3
GCaMP6s	6	10	0.45±0.07	3.4±0.4	0.5±0.2	190±50

^a^ Rise half-time was measured as the time between the stimulus onset and the half-peak of response.

^b^ Decay half-time was calculated as the time from the peak to the half-peak at the end of the response.

^c^ ΔF/F_0_ was calculated as (F − F_0_)/F_0_, where F_0_ is the baseline fluorescence signal averaged over a 1-s period immediately after the start of imaging. 400 and 470 nm excitation light were used for imaging of 402- and 493-form, respectively.

^d^ Signal-to-noise ratio (SNR) was quantified as the peak ΔF/F_0_ response over the standard deviation of the signal during a one-second period before stimulation. Values are shown as the means  ±  standard errors of the mean.

We also estimated linearity of responses elicited by a different number of APs in the neurons expressing FGCaMP using intracellular stimulation with patch clamp or external electric field. We recorded responses to 2–25 APs induced with patch clamp using a frequency of 50 Hz and both 400 and 470 nm excitation light. It was found that in the range of 2 to 25 APs both forms of FGCaMP showed a linear dependence of maximal ΔF/F values from the number of APs (Fig 5D). Stimulation with 5–160 pulses of external electric field (1 ms, 5 V and 87 Hz), resulted in a linear averaged FGCaMP fluorescence responses in the range of 10–80 pulses (Figure M in S1 Supporting Information).

Thus, in neurons, FGCaMP robustly and linearly responded to intracellular or external electric stimulations however with a substantially worse response as compared with GCaMP6s indicator and demonstrated kinetics of association-dissociation with Ca^2+^ ions similar or even faster than that for the GCaMP6s indicator.

### *In vivo* visualization of neuronal activity in larval zebrafish using FGCaMP and wide-field, confocal or light sheet microscopy

To explore the applicability of FGCaMP for *in vivo* recording of neuronal activity, we performed fluorescence imaging of Ca^2+^ activity in a subset of neurons in larval zebrafish at 4 days post fertilization. To deliver the indicators into zebrafish, DNA plasmids encoding FGCaMP or GCaMP6f were co-injected with Tol2 transposase mRNA into the embryos of the pan-neuronal expressing Gal4 zebrafish line. Before imaging, paralyzed larvae were embedded in agarose gel to prevent motion.

**Fig 6:**
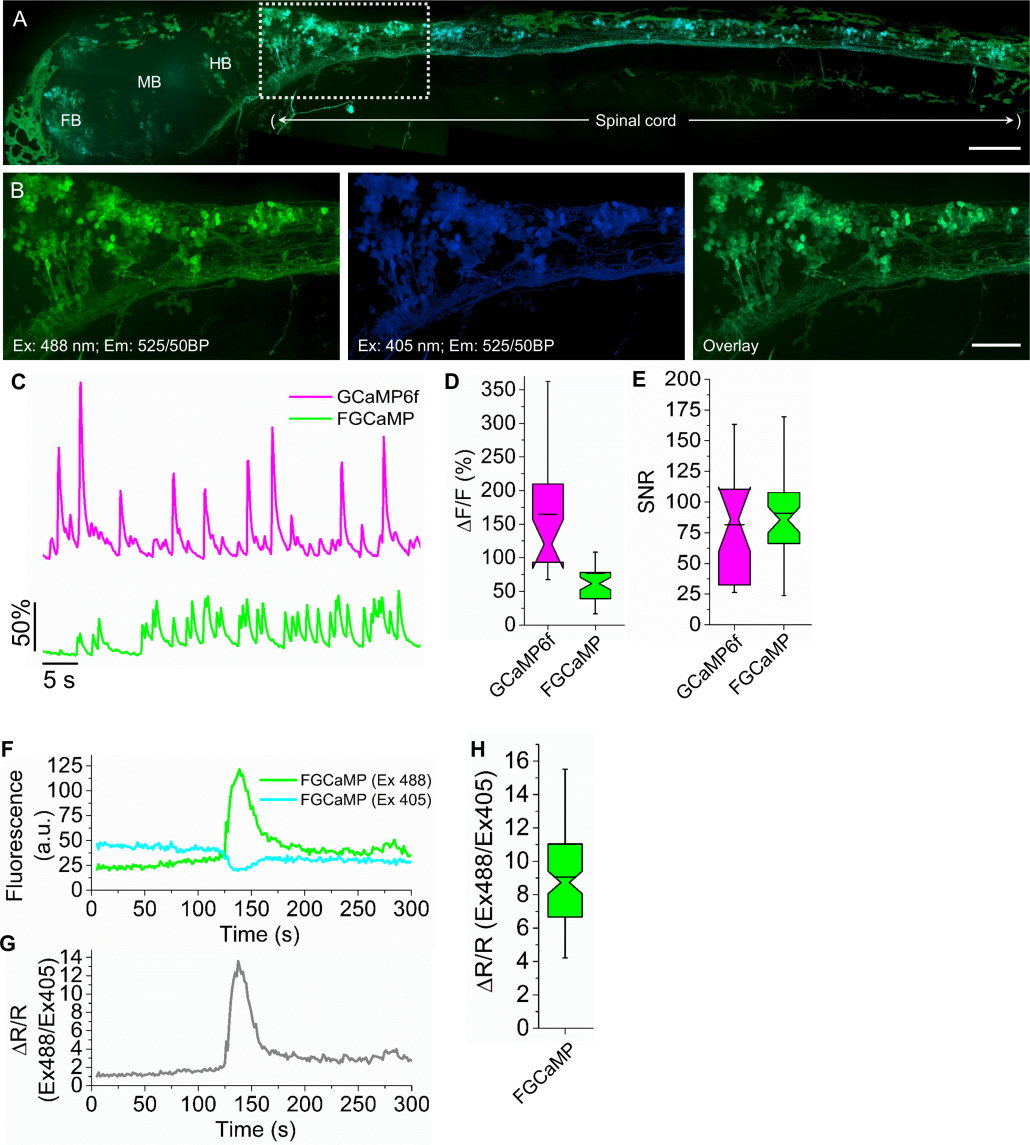
Calcium imaging of neurons expressing FGCaMP in zebrafish larva at 4 days post fertilization. (A) Overlay of confocal fluorescence images of neurons expressing FGCaMP, acquired with 488 and 405 nm excitation and 525/50BP emission. White box indicates area zoomed-in in panel B. Scale bar, 100 μm. FB, forebrain; MB, midbrain; HB, hindbrain. (B) High magnification images of the neurons highlighted in the white box in panel A acquired with 488 nm excitation and 525/50BP emission (*left*; green pseudocolor) and 405 nm excitation and 525/50BP emission (*middle*; blue pseudocolor), and overlay of the left and middle images (*right*). Scale bar, 50 μm. (C) Representative single cell recording of GCaMP6f (*top*; magenta) and FGCaMP green fluorescence responses (*bottom*; green) during 4-aminopyridine induced neuronal activity (Ex: 475/34BP, Em: 527/50BP). Population data for (D) maximum fluorescence changes ΔF/F and (E) SNR corresponding to the experiment in panel C (26 neurons in 3 fish and 43 neurons from 5 fish for GCaMP6f and FGCaMP, respectively). Box plots with notches are used (narrow part of notch, median; top and bottom of the notch, 95% confidence interval for the median; top and bottom horizontal lines, 25% and 75% percentiles for the data; whiskers extend to 5th and 95th percentile for the data; horizontal bar is mean). (F) Representative single cell recording of FGCaMP green fluorescence excited with 488 and 405 nm laser illumination during 4-aminopyridine induced neuronal activity. (G) Fluorescence ratio for the traces shown in panel F. (H) Population data for fluorescence ratio for experiment in panel C (107 neurons in 2 fish). Box plots with notches are used (see panel D for description).

First, we imaged the FGCaMP indicator under a wide-field or confocal microscope with no stimulation. In this conditions both fluorescent 402- and 493-forms of the transiently expressed FGCaMP calcium indicator demonstrated even cytoplasmic localization at the soma and in individual dendrites of neurons localized in FB, MB, HB or spinal cord (Fig 6A and 6B).

Next, we recorded intensiometric fluorescence changes in the green channel (Ex: 475/34BP, Em: 527/50BP) for FGCaMP and control GCaMP6f during 4-aminopyridine (4-AP) induced neuronal activity (Fig 6C). The averaged peak ΔF/F value for FGCaMP was 77%, which was about 2.1-fold lower than that for GCaMP6f (Fig 6D), while the average SNR for FGCaMP was 11% higher than that for GCaMP6f (n = 91 and 82 neurons for FGCaMP and GCaMP6f, respectively; Fig 6E). We noticed that FGCaMP demonstrated high photostability allowing prolonged imaging of neuronal activity.

We further performed ratiometric calcium imaging of 4-AP induced neuronal activity using confocal microscopy. With this aim, we recorded green fluorescence under both 405 and 488 nm laser excitation and 525/50BP emission. As expected from *in vitro* characterization data, during neuronal activity, FGCaMP demonstrated opposing oscillations in green fluorescence intensities when excited with 488 and 405 nm light (Fig 6F, S1 Video). Ratiometric imaging allowed to see resting neurons before activation and also increased the ΔR/R value for FGCaMP.

Finally, we performed a ratiometric calcium imaging of 4-AP induced neuronal activity using light sheet microscopy. Using light sheet microscope we recorded the 4-AP induced activity of ~70 neurons in MB area with 0.7 Hz frequency during 14 min. For registration of 402- and 493-forms we used two 405 and 488 nm light sheets. With 525/50BP emission filter, the average change in the ratio of 488 nm excitation to 405 nm excitation channel was 9-fold (Fig 6G and 6H, S2 Video).

Thus, these data indicate that FGCaMP can reveal chemically-evoked neuronal activity *in vivo* in a model with immobilized larval zebrafish using wide-field, confocal or light sheet microscopy.

## Conclusions

We created calcium indicators with different combinations of CaMs and M13-like peptides or calcineurins from *Aspergillus* fungi and *Komagataella* yeast. From these combinations, we chose the best one and using directed molecular evolution in bacteria, we developed a new genetically encoded cpFP-based green calcium indicator, FGCaMP, with a novel sensory M13-peptide/CaM pair from *Aspergillus* fungi (Fig 1A). We have characterized the main features of this new ratiometric indicator both *in vitro* and *in vivo*. *In vitro*, FGCaMP demonstrates the highest brightness and dynamic range combination among other ratiometric indicators such as GEX-GECO, Pericam and Y-GECO [17,18,19]. It has other beneficial characteristics, such as high photostability, monomeric behavior and fast kinetics of dissociation from Ca^2+^ ions. FGCaMP has a ratiometric phenotype, i.e., upon binding of Ca^2+^ ions the large Stokes shift green fluorescence reduces in its 402-form and increases in its 493-form. In contrast to the intensiometric Ca^2+^ sensors such as GCaMP6 variants and GECOs [16,18], ratiometric changes inthe FGCaMP fluorescence enable quantitative measurements of Ca^2+^ concentrations at around 300 nM in live cells and help to visualize cells both at low and high Ca^2+^ concentrations. Its spectral properties fit the standard excitation sources and filter sets well facilitating its imaging on common fluorescence microscopes in combination with other standard probes such as BFPs, CFPs, and RFPs.

Using site-directed mutagenesis, we found mutations that enhance the fluorescence response and FGCaMP indicator affinity to Ca^2+^ concentration changes of 0–1000 nM that occur during neuronal activity. The FGCaMP indicator and its enhanced versions FGCaMP2 and FGCaMP3 that include a novel sensory part from fungi may be further characterized *in vivo* and used as templates for the development of calcium indicators that have different fluorescent colors and properties.

We further characterized the features of the FGCaMP indicator in the cytoplasm of mammalian cells and on neuronal cultures and demonstrated its novel sensory part provides beneficial high mobility. FGCaMP could reliably visualize variations in Ca^2+^ ion concentrations either induced by ionomycin in mammalian cells or by spontaneous activity in dissociated neuronal cultures. Using FRAP experiments we have found that in contrast to G-GECO1.2 and GCaMP6s standard green GECIs, FGCaMP has high mobility in the mammalian cells at low Ca^2+^ concentrations. This property may be advantageous in terms of cytotoxicity and fluorescence response in living cells.

Using whole-cell patch clamp recording, we revealed faster kinetics of Ca^2+^ responses in neurons expressing FGCaMP compared with those expressing GCaMP6s. We also found that FGCaMP shows a lower sensitivity to APs than GCaMP6s GECI. This low sensitivity correlates with the lower affinity of the FGCaMP indicator to Ca^2+^ ions and with its lower fluorescence responses at low Ca^2+^ concentration changes in the range of 0–1000 nM. Hence, the utilization of enhanced versions of FGCaMP such as FGCaMP2 and FGCaMP3 may address this important limitation.

Finally, we have explored the potential of the FGCaMP indicator for *in vivo* applications. Using wide-field, confocal and light sheet fluorescence microscopy FGCaMP has successfully revealed chemically-evoked neuronal Ca^2+^ ion activity *in vivo* in the nervous system of paralyzed zebrafish. During *in vivo* experiments, the FGCaMP indicator demonstrated a lower ΔF/F dynamic range and revealed less neuronal calcium ion activity than GCaMP6f. Ratiometric *in vivo* imaging with FGCaMP allowed visualization of resting neurons before their activation. Importantly, the ratiometric fluorescence responses of FGCaMP may enable the quantitative estimation of Ca^2+^ ions concentrations.

We believe that further exploration of the novel sensory module from *Aspergillus* fungi and CaMs from other fungi species may provide enhanced sensors that demonstrate superior properties yet are distinct from GECIs with conventional sensory parts from metazoans.

## Materials and methods

### Mutagenesis and library screening

CaMs were cloned from *Aspergillus niger* fungus (F-1057, VKPM, Russia) and *Komagataella pastoris* yeast (Y-3490, VKPM, Russia). mRNAs from fungus and yeast were isolated using RNaesy Plus Mini kit (Qiagen, USA). cDNAs were PCR amplified using reverse transcriptase kit (Evrogen, Russia) with CaMs specific primers (Table C in S1 Supporting Information). M13-like and calcineurin peptides were synthesized from oligonucleotides (Table C in S1 Supporting Information) by polymerase chain reaction (PCR) with overlapping fragments [24].

Primary construction of sensors and directed saturated mutagenesis of linkers between fluorescent and sensory parts were accomplished using PCR with overlapping fragments [24]. For PCR amplification, we used a С1000 Touch Thermal Cycler (Bio-Rad, USA). Random mutations were introduced over the whole length of the sensor gene using PCR in the presence of Mn^2+^ ions with conditions to achieve 2–3 random mutations per 1000 bp according to the Diversify PCR Random Mutagenesis Kit User Manual (Clontech, USA).

Further, we cloned genes for sensors using the BglII/HindIII restriction sites of the pBAD/HisB-TorA plasmid encoding the TorA signal sequence, which is necessary for the transport of sensors into the periplasmic space of bacteria and transformed these plasmids into bacteria. To that end, PCR products were purified in 1% agarose gels and extracted using PCR purification and a gel extraction kit (Evrogen, Russia). Afterward, plasmids and PCR digests were ligated. Ligation mixes were further purified via PCR purification and using a gel extraction kit (Evrogen, Russia) and were transformed into electrocompetent BW25113 bacteria using electroporation.

Screening of bacterial libraries was sequentially performed on Petri dishes, bacterial suspensions in a 96-well plate format, and purified proteins.

Primary screening of approximately 20,000–40,000 colonies of bacterial library expressing calcium sensors variants was performed on Petri dishes under fluorescent stereomicroscope Leica M205FA (Leica, Germany). Expression of the sensors in the colonies on Petri dishes was induced with 0.0002% arabinose for 16 h at 37°C and 24 h at room temperature (r.t.). Reaction of the sensors with calcium ions was further monitored under the fluorescent stereomicroscope Leica. Green fluorescence of both forms was registered by 405/40BP and 480/40BP excitation filters, respectively, and 510/40BP and 535/40BP emission filters, respectively. Fluorescence images of Petri dishes with bacterial colonies were snapped before and after spraying the plates with 100 mM EDTA, 100 mM Na_2_HPO_4_ at pH 7.4. Images obtained were analyzed using the ImageJ software and 60–96 colonies having the highest brightness and contrast were picked up for further analysis on bacterial streaks.

Next, approximately ~30–40 mutants selected through analysis of streaks were analyzed on bacterial suspensions using 96-well Modulus^TM^ II Microplate Reader (Turner Biosystems, USA). For this purpose, the best streaks picked up from Petri dishes were grown in 200 uL aliquots of LB medium containing 100 μg/ml ampicillin, 0.0002% arabinose, and 100 μM CaCl_2_ for 12–16 h at 220 rpm and 37°C and for 24 h at r.t. Bacterial suspensions containing 180 μl of 100 mM NaOAc pH 7.4 buffer supplemented with 100 μM CaCl_2_ and 20 μl of bacterial culture were aliquoted onto 96-well plates. These suspensions were incubated at r.t. for 1 h with measurement of the fluorescence signal. Afterward, EDTA solution was added until a final concentration of 0.4 mM, followed by fluorescence registration for 10 min. Next, a solution of CaCl_2_ was added until a final concentration of 5 mM, followed by fluorescence recording for 10 min. Data collected were analyzed using the Origin 6.0 software, as plots of the dependence of fluorescence vs time. Clones having the highest brightness and contrast in response to the addition of CaCl_2_ and EDTA were selected. On last round of mutagenesis, we skipped the step with analysis on bacterial suspension.

The ~5–10 best clones found on bacterial suspensions or streaks were subsequently grown for protein purification in LB supplemented with 0.0002% arabinose, 100 μg/ml ampicillin and 100 μM CaCl_2_ overnight at 37°C, 220 rpm. The cultures were centrifuged at 1,640 g for 15 min. The cell pellets were resuspended in B-Per solution (Thermo Scientific, USA) containing 1 mg/mL lysozyme and 20 u/mL DNAse I (Invitrogen, USA). The recombinant proteins were purified using Ni-NTA resin (Qiagen, USA). Purified proteins were characterized for brightness (product of quantum yield and extinction coefficient) and contrast as described below. Clones exhibited increased brightness and contrast were compared to clones from the previous round of mutagenesis and subjected to the next round of random mutagenesis.

### Protein purification and characterization

The genes for protein expression were cloned into the pBAD/HisD vector (Invitrogen, USA) at the BglII/HindIII restriction sites, and the resulting plasmids were transformed into BW25113 bacteria. The bacterial cultures were grown in LB medium supplemented with 0.02% arabinose and 100 μg/ml ampicillin overnight at 37°C and 220 rpm. The cultures were then centrifuged at 4648 g for 10 min, and the cell pellets were resuspended in PBS at pH 7.4 with 300 mM NaCl and lysed by sonication on ice. The recombinant proteins were purified using Ni-NTA resin (Qiagen, USA), followed by dialysis for 12–16 h against buffer solutions (30 mM HEPES, 100 mM KCl, pH 7.2, with either 10 mM EDTA (buffer A) or 10 mM CaCl_2_ (buffer B) or without EDTA and CaCl_2_ (buffer C)). The absorbance values and excitation and emission spectra were measured with a CM2203 spectrofluorometer (Solar, Belarus).

Chromophore extinction coefficients for purified FGCaMP in the Ca^2+^-free and Ca^2+^-saturated states were measured in buffers A or B, respectively, by alkaline denaturation with 1 M NaOH and using extinction coefficients for GFP-like chromophores equal to 44,000 M^**−**1^ cm^**−**1^ in 1 M NaOH [25].

For quantum yield determination, the integrated fluorescence values of purified FGCaMP in the Ca^2+^-free and Ca^2+^ saturated states were measured in buffers A or B, respectively, and were compared with equally absorbing GCaMP6s in the saturated state (quantum yield of 0.61 ref. [16]) or mTagBFP2 (quantum yield of 0.64 ref. [26]), as previously reported [27].

Purified FGCaMP (5 mg/ml) protein was diluted 1:5,000 in buffer (30 mM HEPES, 100 mM KCl, pH 7.2) containing 10 mM EGTA (zero free Ca^2+^) or 10 mM Ca-EGTA (39 μM free Ca^2+^). These two stocks were mixed in various ratios to give solutions with different free Ca^2+^ concentrations, as described previously [18]. The *K*_*d*_ and n values were calculated by fitting experimental data with Hill’s equation.

Size-exclusion chromatography was performed with a SuperdexTM 75 10/300 GL column using GE AKTA Explorer (Amersham Pharmacia, UK) FPLC System.

For determination of the pH dependence according to fluorescence, FGCaMP protein was dialyzed in Ca^2+^-free buffer A or in Ca^2+^ containing buffer B. Next, it was diluted 1:100 into a series of pH adjusted buffers (30 mM citric acid, 30 mM borax, and 30 mM NaCl) with pH values ranging from 9 to 3.5 in 0.5 pH units interval in a 96-well black clear bottom plate (Thermo Scientific, USA), as described in the original paper [18]. Fluorescence was measured using a Modulus^TM^ II Microplate Reader (TurnerBiosystems, USA) and CM2203 spectrofluorometer (Solar, Belarus).

For determination of the pH dependence according to absorbance, FGCaMP protein was dialyzed in 100mM KCl, pH ~7.5 supplemented with either 20 mM CaCl_2_ or 10 mM EDTA. Next, it was diluted 1:10 into a series of pH adjusted buffers (30 mM citric acid, 30 mM borax, and 30 mM NaCl) with pH values ranging from 10 to 5 in 0.5 pH units interval in a 1.5 ml tubes. Upon incubation at r.t. for 20 min, absorbance was measured using a NanoDrop 2000c spectrophotometer (Thermo Scientific, USA).

Photobleaching experiments were performed with suspensions of purified proteins in mineral oil, as previously described [28]. Briefly, the kinetics of photobleaching was measured using purified proteins dialyzed in Ca^2+^-free buffer A or in Ca^2+^ containing buffer B at a 1 mg/ml concentration, in aqueous microdroplets in mineral oil using Zeiss Axio Imager Z2 microscope (Zeiss, Germany) equipped with a 120 W mercury short-arc lamp (LEJ, Germany), a 63x 1.4 NA oil immersion objective lens (PlanApo, Zeiss, Germany), a 355/25 BP and 470/40BP excitation filter, a FT 495 beam splitter, and 525/50BP emission filter. Light power density was measured at a rear focal plane of the objective lens. The times to photobleach from 1000 down to 500 emitted photons per second were calculated according to standard procedures [20]. In brief, the averaged raw data were corrected for a spectral output of the lamp, transmission profiles of the excitation filter and dichroic mirror, and absorbance spectra of the respective green fluorescent proteins and their quantum yields. EGFP protein that has been characterized according to this procedure in [20] was used as a reference for the FGCaMP form absorbing at 493 nm. mTagBFP protein was used as a reference for the FGCaMP form absorbing at 402 nm.

To study protein maturation, BW25113 bacteria transformed with the pBAD/HisB-TorA-FGCaMP plasmid were grown in LB medium supplemented with ampicillin at 37°C overnight. The next morning 0.2% arabinose was added to bacterial cells. Upon induction of the protein expression, the bacterial cultures were grown at 37°C in 50 ml tubes filled to the brim and tightly sealed to restrict oxygen supply. After 2 hours, the cultures were centrifuged in the same tightly closed tubes. After opening the tubes, the bacteria were sonicated in PBS buffer and the resulting proteins were purified using Ni-NTA resin within 10 min, with all procedures and buffers at or below 4°C. Protein maturation of FGCaMP occurred in Ca^2+^ containing buffer B at 37°C. Green fluorescence signal of the protein was monitored using a CM2203 spectrofluorometer (Solar, Belarus) at 402 nm excitation.

### Stopped-flow fluorimetry

The kinetic curves of Ca^2+^-association with FGCaMP and GCaMP6s were acquired on a Chirascan Spectrofluorimeter equipped with a stopped-flow module (Applied Photophysics, UK). Ca^2+^ (varying concentrations) and protein solutions (20 μg/ml in 30 mM HEPES buffer (pH 7.2) containing 100 mM KCl) were prepared as described in [5]. Fluorescence excitation was set to 500 nm for GCaMP6s and to 500 or 402 nm for FGCaMP. Fluorescence emission was detected using a 515 nm cut-off filter. Exponential fitting of the fluorescence signal changes over time and fitting the observed data to the equation k_obs_ = k_on_× [Ca^2+^]^n^+ k_off_ were performed as described in [5]. In the Ca^2+^- dissociation kinetics experiments, protein solution (20 μg/ml) in 30 mM HEPES (pH 7.2), 100 mM KCl and 1 μM CaCl_2_ (or 0.35 μM CaCl_2_ for FGCaMP) was rapidly mixed (1:1) with 30 mM HEPES (pH 7.2), 100 mM KCl, and 10 mM EGTA.

### Mammalian plasmids construction

In order to construct the pAAV-*CAG*-R-GECO1 plasmid R-GECO1 was PCR amplified as KpnI-EcoRI fragment and swapped with the iRFP-P2A-EGFP gene in the pAAV-*CAG-*iRFP-P2A-EGFP vector. In order to construct pAAV-*CAG*-FGCaMP and pAAV-*CAG*-GCaMP6s plasmids FGCaMP and GCaMP6s were PCR amplified as BamHI-HindIII and BamHI-EcoRI fragments and swapped with the TagRFP gene in the pAAV-*CAG-*TagRFP vector. In order to construct the pAAV-*CAG*-TagRFP plasmid, the TagRFP (Evrogen) gene was PCR amplified as NheI-EcoRI fragment and swapped with the iRFP-P2A-EGFP gene in the pAAV-*CAG-*iRFP-P2A-EGFP vector.

For transient expression in zebrafish larvae, we designed expression vector by cloning the 4 non-repetitive upstream activating sequences (4nrUAS) as previously described [29] together with a beta actin core minimal promoter followed by the 1.2 kb long 3'UTR sequence of *Danio rerio* synaptotagmin IIa (syt2a). The expression cassette was flanked by Tol2 transposon ends. FGCaMP and GCaMP6f genes were PCR amplified and cloned into the designed pTol2-4nrUAS vector using *Spe*I and *Asc*I sites.

### Cell culture and transfection

HeLa Kyoto cells were maintained in Dulbecco’s Modified Eagle Medium (DMEM) (GIBCO) supplemented with 10% fetal bovine serum (FBS) (Sigma), 2 mM GlutaMax-I (GIBCO), 50 U/ml penicillin, and 50 μg/ml streptomycin (GIBCO). Plasmids for transfection were prepared using a Plasmid Miniprep purification kit (Evrogen, Russia). Transfection was performed using TurboFectTM (Thermo Fisher Scientific, USA) according to the manufacturer’s protocol.

### rAAV particle production and isolation

The rAAV particles were purified as described [5]. HEK293T cells were kindly provided by Chumakov P.M. from Moscow, IMB.

### Mammalian live-cell imaging

HeLa Kyoto cell (kindly gifted by Belousov V.V from Moscow, IBC) cultures were imaged 24–48 h after transfection using a laser spinning-disk Andor XDi Technology Revolution multi-point confocal system (Andor, UK) equipped with an inverted Nikon Eclipse Ti microscope, a 75 W mercury-xenon lamp (Hamamatsu, Japan), a 60× oil immersion objective (Nikon, Japan), a 16-bit QuantEM 512SC electron-multiplying CCD (Photometrics, USA), and a cage incubator (Okolab, Italy). Before imaging, the culture medium was changed to Dulbecco’s Phosphate Buffered Saline (DPBS) buffered with 20 mM HEPES, pH 7.4.

For time-lapse imaging experiments with varying Ca^2+^ concentration, 1 mM EDTA and 5 μM ionomycin were added to cells for imaging calcium sensors in the Ca^2+^-free state. After imaging calcium indicators in the apo-state, cells were washed with DPBS buffered with 20 mM HEPES, pH 7.4. Next, 2 mM CaCl_2_ and 5 μM ionomycin were added to induce fluorescence signal for Ca^2+^-saturated calcium indicators.

### FRAP (fluorescence recovery after photobleaching) experiments

FRAP experiments were carried out in HeLa Kyoto cells transiently transfected with FGCaMP and control GECIs (GCaMP6s, and G-GECO1.2) at physiological and elevated Ca^2+^ concentrations. Each FRAP-experiment lasts for 15–20 minutes with cells kept in an incubator at 37°C, 5% CO_2_ and high humidity. Experiments were done with the LSM780 system (Carl Zeiss, Germany).

FRAP experiments were done in “spot” mode with a 488-nm laser for bleaching and data acquisition. 100x objective (NA = 1.46 oil immersion) was used in all experiments and pinhole was fully opened.

Laser intensity (between 0.05–0.2 μW) and detector gain were adjusted to get maximum signal without detector oversaturation to maximize dynamic range. Spectral range of detector was set to 490–570 nm.

Fluorescence recovery was traced for 600 ms after 40 ms (Fig 4C–4F) or 310 ms (Figure L in S1 Supporting Information) of bleaching with 100% laser power (~50 μW). Bleaching duration was chosen to have no impact on recovery rate. Bleaching was started after reaching of constant fluorescence signal.

The FRAP curve was treated as follows: maximum intensity (before bleaching) was normalized to 1 a.u., and minimal intensity (immediately after bleaching) was normalized to 0 a.u. FRAP curves from 5 different cells were averaged to produce each FRAP curve with standard deviations shown on Fig 4. Percent of the mobile fraction was found by dividing mean intensity at the end of recovery over mean intensity before bleaching.

For experiments at elevated Ca^2+^ concentrations, 2 mM CaCl_2_ and 5 μM ionomycin were added to the cells. For experiments concerning reversibility of mobility, FRAP measurements were performed sequentially at elevated Ca^2+^ concentrations (in the presence of 2 mM CaCl_2_ / 5 μM ionomycin) and upon elimination of Ca^2+^ ions (upon washing of the cells and addition of 1mM EDTA / 5 μM ionomycin).

### Isolation, transduction, transfection, and imaging of neuronal cultures

Dissociated neuronal cultures were isolated from C57BL/6 mice at postnatal days 0–3 and were cultivated on 35-mm MatTek glass-bottom dishes in a full Neurobasal medium, i.e. Neurobasal Medium A (GIBCO, UK) supplemented with 2% B27 Supplement (GIBCO, UK), 0.5 mM GlutaMax-I (GIBCO, UK), 50 U/ml penicillin, and 50 μg/ml streptomycin (GIBCO, UK). On the 4^th^ day *in vitro* (DIV), neuronal cells were transduced with 1–2 μl rAAV viral particles (titer of ~0.5^10^6^ particles/μl) carrying AAV-*CAG*-FGCaMP or AAV-*CAG*-R-GECO1. On the day 14–21^st^ after transduction cells were imaged using an Andor XDi Technology Revolution multi-point confocal system.

On the 5–7^th^ DIV neuronal cultures were transfected by Ca^2+^-phosphate method with some modifications [30]. DNA plasmids were isolated using a Plasmid Midiprep purification kit (Evrogen, Russia). DNAs were mixed with 2 M CaCl_2_ and 2xHBSS buffer and after incubation at r.t. for 10 min formed DNA pellet was added to the neuronal culture. After 20–60 min of incubation at 37°C and 5% CO_2_, neuronal cultures were washed with MEM medium (GIBCO, UK) supplemented with 20 mM HEPES (pH 6.8). Washing was repeated till complete disappearance of DNA pellet (usually two washing were enough). Each washing lasted for 4 min with incubation at 37°C and 5% CO_2_. Finally, cultures were incubated in the full Neurobasal medium. One-two days after transfection cultures were imaged using an Andor XDi Technology Revolution multi-point confocal system.

### Stimulation of neuronal cultures with electric field

Stimulation of neuronal cultures with the electric field was performed according to described protocol [16]. Briefly, transfected neurons expressing FGCaMP were stimulated in the full Neurobasal medium supplemented with a 10 μM 6-Cyano-2,3-dihydroxy-7-nitro-quinoxaline (CNQX) and 100 μM 2-Amino-5-phosphonovalerate (APV) to inhibit spontaneous activity. Action potentials (APs) were evoked by field stimulation with a custom-built stimulation unit and a custom-built 35 mm cap stimulator with pairs of parallel platinum-iridium wires. Individual pulses of 1 ms, 5 V, and 87 Hz reliably triggered APs. Fluorescence changes were monitored using Andor XDi Technology Revolution multi-point confocal system.

### Whole-cell electrophysiology and calcium imaging

Whole-cell recordings with patch electrodes were made from cultured neurons, expressing GECIs. Cells were selected under visual control using 470/20 BP and 510LP green fluorescence filter set, Nomarski optics, and infrared video microscopy. The patch electrodes were filled with a potassium gluconate-based solution (130 mM potassium gluconate, 20 mM KCl, 4 mM Mg-ATP, 0.3 mM Na_2_-GTP, 10 mM sodium phosphocreatine, 10 mM HEPES at pH 7.3) and had a resistance of 6–8 MΩ. During recording, cells were bathed in modified Hank’s solution containing: 138 mM NaCl, 1.26 mM CaCl_2_, 0.5 mM MgCl_2_, 0.4 mM MgSO_4_, 5.3 mM KCl, 0.44 mM KH_2_PO_4_, 4.16 mM NaHCO_3_, 0.34 mM Na_2_HPO_4_, 10 mM Glucose, 10 mM HEPES at pH 7.4 and room temperature. Recordings were made with a MultiClamp 700B (Molecular Devices, USA) amplifier in the bridge mode. After amplification and low-pass filtering at 10 kHz, data were digitized at 20 kHz and fed into a computer using the Digidata 1500 interface and PCLAMP software (Molecular Devices, USA). Cells were stimulated with 50 Hz trains of short (5 ms) intracellularly applied current pulses; the intensity of the pulses was adjusted to reliably induce action potentials for each cell.

Optical imaging was performed on an Olympus BX51WI microscope equipped with UV-compatible 40× water immersion objective, two camera ports, and two collimated light emitting diodes (LED) with the peak emission wavelength of 400 nm and 470 nm (Thorlabs, USA) for epi-illumination. Imaging was performed with a NeuroCCD camera (80 × 80 pixels, RedShirtImaging, USA) using a frame rate of 40 Hz. Fluorescence changes were measured with single wavelength excitation (400 or 470 nm) and emission >510 nm. Analysis of optical data, including spatial averaging, high-pass and low-pass filtering, was conducted with the Neuroplex 7 software (RedShirtImaging, USA). The time-courses of the responses were corrected for bleaching using a linear regression computed through the mean values 2 seconds before the stimulation and by subtracting the extrapolated values.

### Transient expression in zebrafish embryos

All experiments with zebrafish were conducted in accordance with MIT Committee on Animal Care. Zebrafish were raised and bred at 28°C according to standard methods. DNA plasmids encoding FGCaMP or GCaMP6f were co-injected with Tol2 transposase mRNA into the embryos of the pan-neuronal expressing Gal4 line, tg(*elavl3*:*GAL4-VP16)*^*nns6*^ [31]. The embryos used in the study were homozygous nacre. Briefly, DNA and Tol2 transposase mRNA, synthesized using pCR2FA as a template [32] (mMESSAGE mMACHINE® SP6 Transcription Kit, Thermofisher), were diluted to a final concentration of 25 ng/μl in 0.4 mM KCl solution containing 0.05% phenol red solution (Sigma Aldrich) to monitor the injection quality. The mixture was kept on ice to minimize degradation of mRNA during the injection. The mixture was injected into embryos at 1–4 cell stages as described previously [33]. Larvae were screened for green fluorescence in the brain and spinal cord at 2–3 days post fertilization (dpf; animals were used without regard to sex) and subsequently imaged at 4 dpf.

### Calcium imaging in zebrafish embryos

Zebrafish larvae at 4 dpf were used to image neurons expressing FGCaMP or GCaMP6f. To prevent motion artifacts during imaging, larvae were paralyzed by applying a paralytic agent, pancronium bromide, at a final concentration of 0.20 mg/ml (Sigma Aldrich), to stop muscle motion [34]. Paralyzed larvae were immobilized in 1.5% ultra-low-melting agarose (Sigma Aldrich) prepared in E3 medium following standard protocols [35]. For neuronal activity imaging, embedded larval zebrafish was stimulated by administration of 4-aminopyridine (Sigma) at final concentration 1 mM. For wide-field calcium imaging, the embedded larvae were mounted on an inverted epifluorescent Nikon Eclipse *Ti* microscope equipped with 40x NA 1.15 (Nikon) objective lens, an SPECTRA-X light engine (Lumencor), and a 5.5 Zyla camera (Andor), controlled by NIS-Elements AR software. The fluorescence of FGCaMP and GCaMP6f was excited with 475/34BP from a LED and imaged with 527/50BP emission filter. Confocal imaging was performed on an inverted Nikon Eclipse Ti microscope equipped with spinning disk sCSU-W1 confocal scanner unit (YOKOGAWA), 405 and 488 nm solid state lasers, 40x NA 1.15 and 20x NA 0.75 (Nikon) objective lenses, and a 4.2PLUS Zyla camera (Andor), controlled by NIS-Elements AR software. Lightsheet imaging for **Supplementary Video 2** was performed on a Zeiss Z.1 light sheet microscope. For image acquisition, paralyzed larvae embedded in ultra-low-melting agarose gel were extruded from the glass capillary and mounded on coverslip. Light sheets were generated by two illumination objectives (10x, NA 0.2) with 405 and 488 nm laser excitation, and the fluorescence signal detected by a 20x water immersion objective (NA 1.0). Both light sheets were used for data collection.

### Animal care

All experiments with mice were approved by the National Research Center “Kurchatov Institute” Committee on Animal Care (protocol No. 1, 7 September 2015) and were in accordance with the *Russian* Federation Order Requirements N *267* МЗ and the National Institutes of Health Guide for the Care and Use of Laboratory Animals. Ten C57BL/6 mice were used in this study, ages P0 old. For euthanasia of P0 neonates, we used decapitation by sharp scissors. Mice were used without regard to gender.

## Supporting information

S1 Supporting information **File containing all supporting Figures (A-O) and Tables (A-C). Figure A in S1 Supporting information.** Alignment of the amino acid sequences for CaMs and peptides from CaM-dependent kinases and calcineurins found in different species. **Figure B in S1 Supporting information.** Alignment of the amino acid sequences for original library and FGCaMP calcium indicator. **Figure C in S1 Supporting information.** pH dependence of absorbance for the purified FGCaMP indicator. **Figure D in S1 Supporting information.** Size-exclusion chromatography for FGCaMP protein. **Figure E in S1 Supporting information.** Ca^2+^ titration curves for FGCaMP and GCaMP6s in the absence or presence of 1 mM MgCl_2_. **Figure F in S1 Supporting information.** Kinetic curves obtained from Ca^2+^-association experiments. **Figure G in S1 Supporting information.** Detailed analysis of the onset kinetics for Ca^2+^-binding. **Figure H in S1 Supporting information.** Alignment of the amino acid sequences of M13-like peptides and CaMs from FGCaMP and the other calcium indicators. **Figure I in S1 Supporting information.** Ca^2+^ titration curves for FGCaMP mutants. **Figure J in S1 Supporting information.** Ca^2+^ titration curves for FGCaMP2 mutants with mutations in CaM part. **Figure K in S1 Supporting information.** Ca^2+^ titration curves for FGCaMP3 variants with mutations in the M13-like peptide. **Figure L in S1 Supporting information.** Reversible changes in the mobile fraction of the FGCaMP indicator at the transition from high to low Ca^2+^ concentrations in HeLa Kyoto cells studied by FRAP experiments. **Figure M in S1 Supporting information.** Fluorescence changes in response to the external electric field in cultured neurons expressing FGCaMP indicator. **Figure N in S1 Supporting information.** Confocal ratiometric calcium imaging with FGCaMP during 4-AP induced neuronal activity in paralyzed larvae embedded in ultra-low-melting agarose gel. **Figure O in S1 Supporting information.** Lightsheet ratiometric calcium imaging with FGCaMP during 4-AP induced neuronal activity in paralyzed larvae embedded in ultra-low-melting agarose gel. **Table A in S1 Supporting information.** Brightness and contrasts for FGCaMP and other ratiometric green and yellow GECIs. **Table B in S1 Supporting information.** K_d_ values and fluorescence contrasts for 402- and 493-forms of FGCaMP and its mutants. **Table C in S1 Supporting information.** List of primers.
(PDF)

S1 Dataset Excel file containing values of all data points presented in this manuscript. (XLSX)

S1 Video Confocal ratiometric calcium imaging with FGCaMP during 4-AP induced neuronal activity in paralyzed larvae embedded in ultra-low-melting agarose gel. (AVI)

S2 Video Lightsheet ratiometric calcium imaging with FGCaMP during 4-AP induced neuronal activity in paralyzed larvae embedded in ultra-low-melting agarose gel. (AVI)
