# Supplementary material for: Green fluorescent genetically encoded calcium indicator based on calmodulin/M13-peptide from fungi
Source: PLoS One. 2017 Aug 24;12(8):e0183757. doi: 10.1371/journal.pone.0183757 (PMC5570312; doi:10.1371/journal.pone.0183757)
Supplement: S1 Supporting information — File containing all supporting Figures (A-O) and Tables (A-C). Figure A in S1 Supporting information. Alignment of the amino acid sequences for CaMs and peptides from CaM-dependent kinases and calcineurins found in different species. Figure B in S1 Supporting information. Alignment of the amino acid sequences for original library and FGCaMP calcium indicator. Figure C in S1 Supporting information. pH dependence of absorbance for the purified FGCaMP indicator. Figure D in S1 Supporting information. Size-exclusion chromatography for FGCaMP protein. Figure E in S1 Supporting information. Ca2+ titration curves for FGCaMP and GCaMP6s in the absence or presence of 1 mM MgCl2. Figure F in S1 Supporting information. Kinetic curves obtained from Ca2+-association experiments. Figure G in S1 Supporting information. Detailed analysis of the onset kinetics for Ca2+-binding. Figure H in S1 Supporting information. Alignment of the amino acid sequences of M13-like peptides and CaMs from FGCaMP and the other calcium indicators. Figure I in S1 Supporting information. Ca2+ titration curves for FGCaMP mutants. Figure J in S1 Supporting information. Ca2+ titration curves for FGCaMP2 mutants with mutations in CaM part. Figure K in S1 Supporting information. Ca2+ titration curves for FGCaMP3 variants with mutations in the M13-like peptide. Figure L in S1 Supporting information. Reversible changes in the mobile fraction of the FGCaMP indicator at the transition from high to low Ca2+ concentrations in HeLa Kyoto cells studied by FRAP experiments. Figure M in S1 Supporting information. Fluorescence changes in response to the external electric field in cultured neurons expressing FGCaMP indicator. Figure N in S1 Supporting information. Confocal ratiometric calcium imaging with FGCaMP during 4-AP induced neuronal activity in paralyzed larvae embedded in ultra-low-melting agarose gel. Figure O in S1 Supporting information. Lightsheet ratiometric calcium imaging with FGCaMP during 4-AP induced n [file pone.0183757.s001.pdf]

Supporting information for:

Green fluorescent genetically encoded calcium indicator based on calmodulin/M13-peptide from fungi

Natalia V. Barykina[1,2], Oksana M. Subach[1,3], Kiryl D. Piatkevich[4], Erica E. Jung[4], Aleksey Y. Malyshev[5], Ivan V. Smirnov[5,6], Andrey O. Bogorodskiy[1], Valentin I. Borshchevskiy[1], Anna M. Varizhuk[7,8], Galina E. Pozmogova[7], Edward S. Boyden[4,9], Konstantin V. Anokhin[2,3,10], Grigori N. Enikolopov[1,11,12*], & Fedor V. Subach[1*]

[1]Moscow Institute of Physics and Technology, Dolgoprudny, Moscow Region, Russia
[2]P.K. Anokhin Institute of Normal Physiology of RAMS, Moscow, Russia
[3]National Research Center "Kurchatov Institute", Moscow, Russia
[4]MIT Media Lab, Massachusetts Institute of Technology, Cambridge, MA, USA
[5]Institute of Higher Nervous Activity and Neurophysiology of RAS, Moscow, Russia
[6]Medico-Biological Faculty, N.I. Pirogov Russian National Research Medical University, Moscow, Russia
[7]Federal Research and Clinical Center of Physical-Chemical Medicine of Federal Medical Biological Agency, Moscow, Russia
[8]Engelhardt Institute of Molecular Biology RAS, Moscow, Russia
[9]MIT McGovern Institute for Brain Research, MIT, Cambridge, MA, USA
[10]Lomonosov Moscow State University, Moscow, Russia
[11]Department of Anesthesiology, Stony Brook University Medical Center, NY, USA
[12]Center for Developmental Genetics, Stony Brook University, NY, USA

*Correspondence and requests for materials should be addressed to F.V.S. (email: subach.fv@mipt.ru) or G.N.E. (email: Grigori.Enikolopov@stonybrookmedicine.edu)

S1 Supporting information

Figure A. Alignment of the amino acid sequences for CaMs and peptides from CaM-dependent kinases and calcineurins found in different species.

Figure B. Alignment of the amino acid sequences for original library and FGCaMP calcium indicator.

Figure C. pH dependence of absorbance for the purified FGCaMP indicator.

Figure D. Size-exclusion chromatography for FGCaMP protein.

Figure E. Ca^{2+} titration curves for FGCaMP and GCaMP6s in the absence or presence of 1 mM $MgCl_2$.

Figure F. Kinetic curves obtained from Ca^{2+}-association experiments.

Figure G. Detailed analysis of the onset kinetics for Ca^{2+}-binding.

Figure H. Alignment of the amino acid sequences of M13-like peptides and CaMs from FGCaMP and the other calcium indicators.

Figure I. Ca^{2+} titration curves for FGCaMP mutants.

Figure J. Ca^{2+} titration curves for FGCaMP2 mutants with mutations in CaM part.

Figure K. Ca^{2+} titration curves for FGCaMP3 variants with mutations in the M13-like peptide.

Figure L. Reversible changes in the mobile fraction of the FGCaMP indicator at the transition from high to low Ca^{2+} concentrations in HeLa Kyoto cells studied by FRAP experiments.

Figure M. Fluorescence changes in response to the external electric field in cultured neurons expressing FGCaMP indicator.

Figure N. confocal ratiometric calcium imaging with FGCaMP during 4-AP induced neuronal activity in paralyzed larvae embedded in ultra-low-melting agarose gel.

Figure O. Lightsheet ratiometric calcium imaging with FGCaMP during 4-AP induced neuronal activity in paralyzed larvae embedded in ultra-low-melting agarose gel.

Table A. Brightness and contrasts for FGCaMP and other ratiometric green and yellow GECIs.

Table B. K_d values and fluorescence contrasts for 402- and 493-forms of FGCaMP and its mutants.

Table C. List of primers.

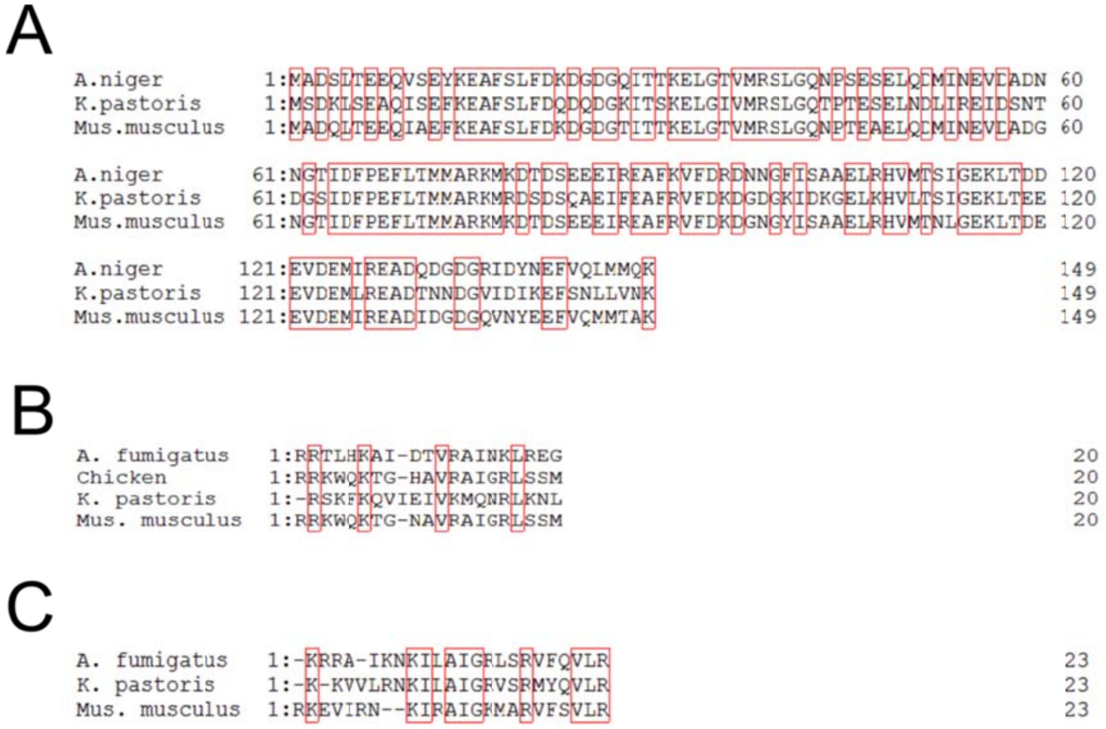

Figure A. Alignment of the amino acid sequences for CaMs and peptides from CaM-dependent kinases and calcineurins found in different species. (A) Alignment of the amino acid sequences for CaMs from *A. niger*, *K. pastoris*, and *M. musculus*. (B) Alignment of the amino acid sequences for M13-like peptides from CaM-dependent kinases from *A. fumigatus*, chicken, *K. pastoris*, and *M. musculus*. (C) Alignment of the amino acid sequences for calcineurins from *A. fumigatus*, *K. pastoris*, and *M. musculus*. Identical residues are selected with red boxes.

```
                -1        10        20        30        40        50        60
                 |         |         |         |         |         |         |
Library         MRRTLHKAIDTVRAINKLREGXXNVYIKADKQKNGIKANFHIRHNIEDGGVQLAYHYQQNT
FGCaMP          MRRTLHKAIDTVRAINKLREGLGNVYIKADKQKNGIKANFKIRHNIEDGGVQLAYHYQQNT

                          70        80        90       100       110       120
                           |         |         |         |         |         |
Library          PIGDGPVLLPDNHYLSVQSKLSKDPNEKRDHMVLLEFVTAAGITLGMDELYKGGTGGSMV
FGCaMP           PIGDGPVLLPDNHYLSVQSKLSKDPNEKRDHMVLLEFVTAAGITLGMDELYKGGTGGSMV

                         130       140       150       160       170       180
                           |         |         |         |         |         |
Library          SKGEELFTGVVPILVELDGDVNGHKFSVSGEGEGDATYGKLTLKFICTTGKLPVPWPTLV
FGCaMP           SKGEELFTGVVPILVELDGDVNGHKFSVSGEGEGDATYGKLTLKFICTTGKLPVPWPTLV

                         190       200       210       220       230       240
                    ***    |         |         |         |         |         |
Library          TTLTYGVQCFSRYPDHMKQHDFFKSAMPEGYIQERTIFFKDDGNYKTRAEVKFEGDTLVN
FGCaMP           TTLTYGVQCFSRYPDHMKQHDFFKSAMPEGYIQERTIFFKDDGYYKTRAEVKFEGDTLVN

                         250       260       270       280       290       300
                           |         |         |         |         |         |
Library          RIELKGIDFKEDGNILGHKLEYNXXXMADSLTEEQVSEYKEAFSLFDKDGDGQITTKELG
FGCaMP           RIELKGIDFKEDGNILGHKLEYDVTLMADSLTEEQVSEYKEAFSLFDKDGDGQITTKELG

                         310       320       330       340       350       360
                           |         |         |         |         |         |
Library          TVMRSLGQNPSESELQDMINEVDADNNGTIDFPEFLTMMARKMKDTDSEEEIREAFKVFD
FGCaMP           TVMRSLDQNPSESELQDMINEVDADNNGTIDFPEFLTMMARKMKDTDSEEEIREACKVFD

                         370       380       390       400       410
                           |         |         |         |         |
Library          RDNNGFISAAELRHVMTSIGEKLTDDEVDEMIREADQDGDGRIDYNEFVQLMMQK
FGCaMP           RDNNGFISAAELRHVMTSIGEKLTDDEVDEMIREADQDGDGRIDYNEFVQLMMQK
```

Figure B. Alignment of the amino acid sequences for original library and FGCaMP calcium indicator. Alignment numbering follows that of FGCaMP. Residues from fluorescent part buried in β-can are highlighted with green. Residues that are forming chromophore are marked with asterisks. EF1-EF4 hands responsible for calcium binding are highlighted with cyan color. Mutations in final FGCaMP protein are highlighted with red.

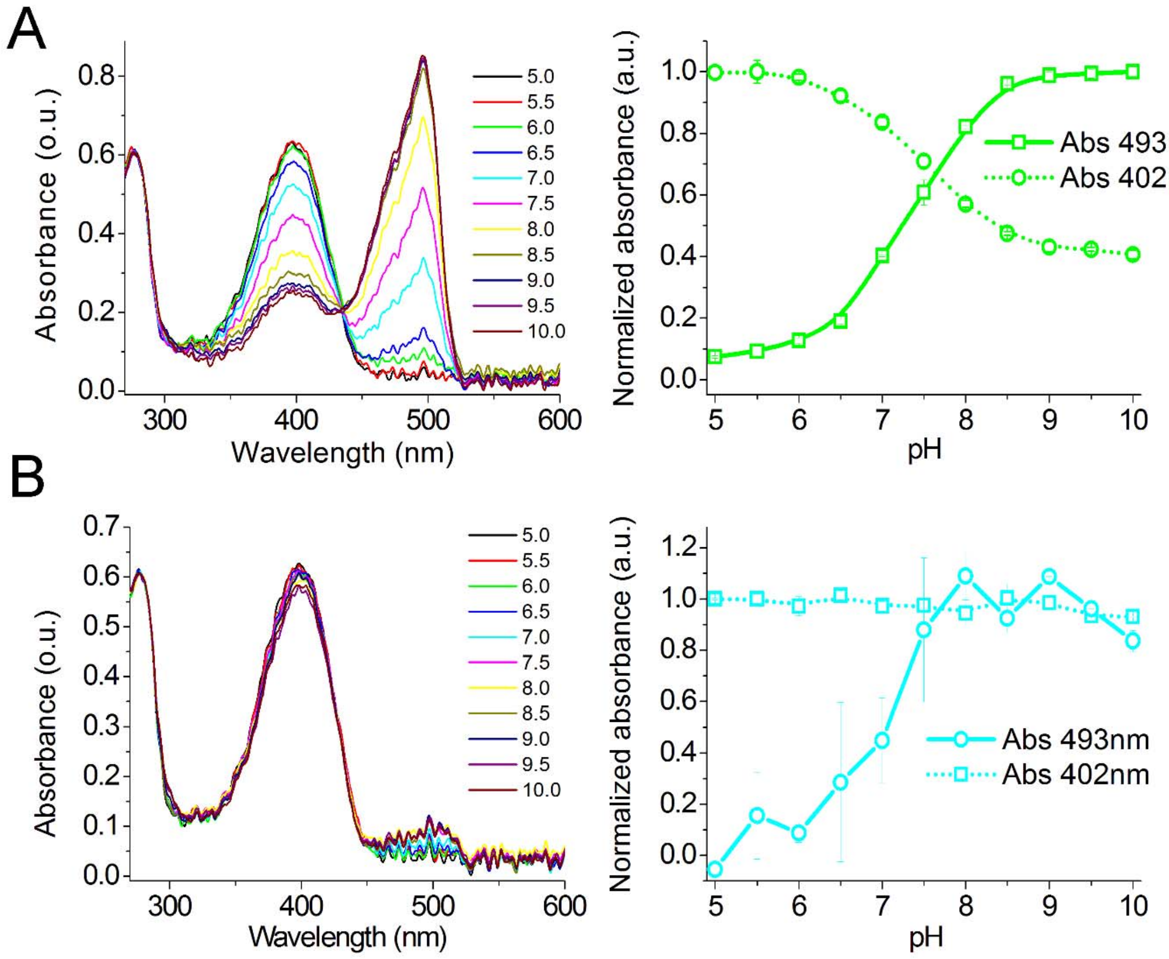

Figure C. pH dependence of absorbance for the purified FGCaMP indicator. (A, B) Absorbance spectra (left) and the normalized absorbance at 402 and 493nm (right) for FGCaMP in Ca^{2+}-bound (A) and Ca^{2+}-free (B) states in the range of pH values of 5-10. Error bars represent the standard deviation.

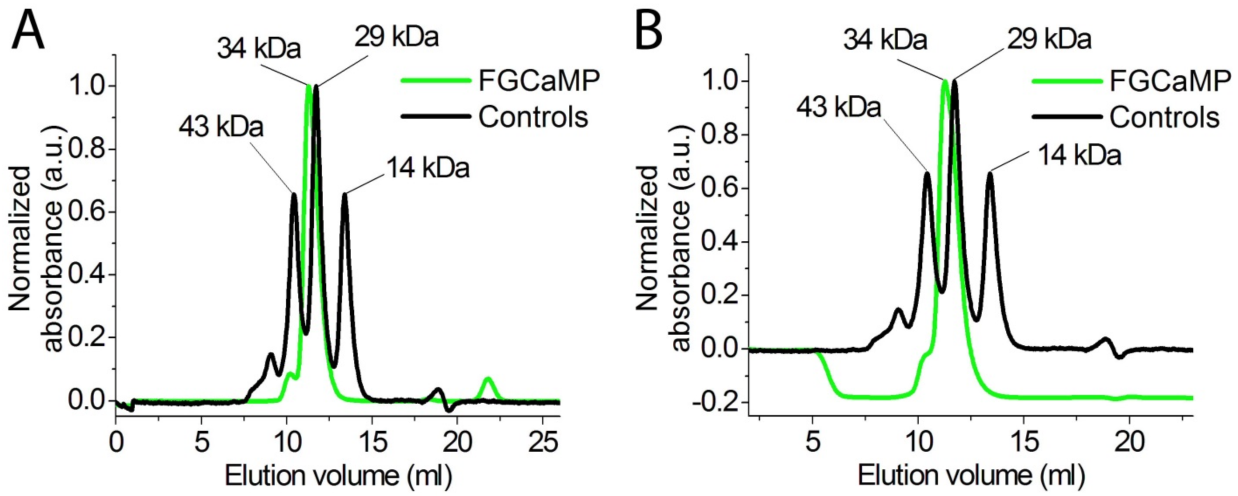

Figure D. Size-exclusion chromatography for FGCaMP protein. Fast protein liquid chromatography of FGCaMP in 20 mM Tris-HCl (pH 7.5), 200 mM NaCl buffer supplemented with 2 mM EDTA (A) or in 20 mM Tris-HCl (pH 7.5), 100 mM NaCl buffer supplemented with 1 mM $CaCl_2$ (B).

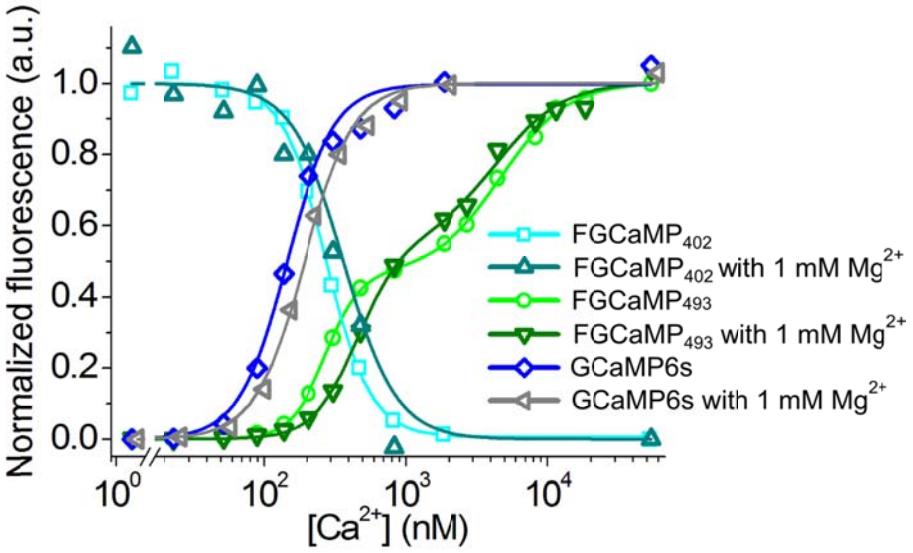

Figure E. Ca^{2+} titration curves for FGCaMP and GCaMP6s in the absence or presence of 1 mM $MgCl_2$. Ca^{2+} titration curves for 402- and 493-forms of FGCaMP were acquired using 402 and 490 nm excitation light, respectively. Fluorescence changes were normalized to maximal and minimal values. Experimental data were fitted to Hill equation or double Hill equation $y = V_1*x^{n1}/(K_{d1}^{n1}+x^{n1}) + V_2*x^{n2}/(K_{d2}^{n2}+x^{n2})$, respectively.

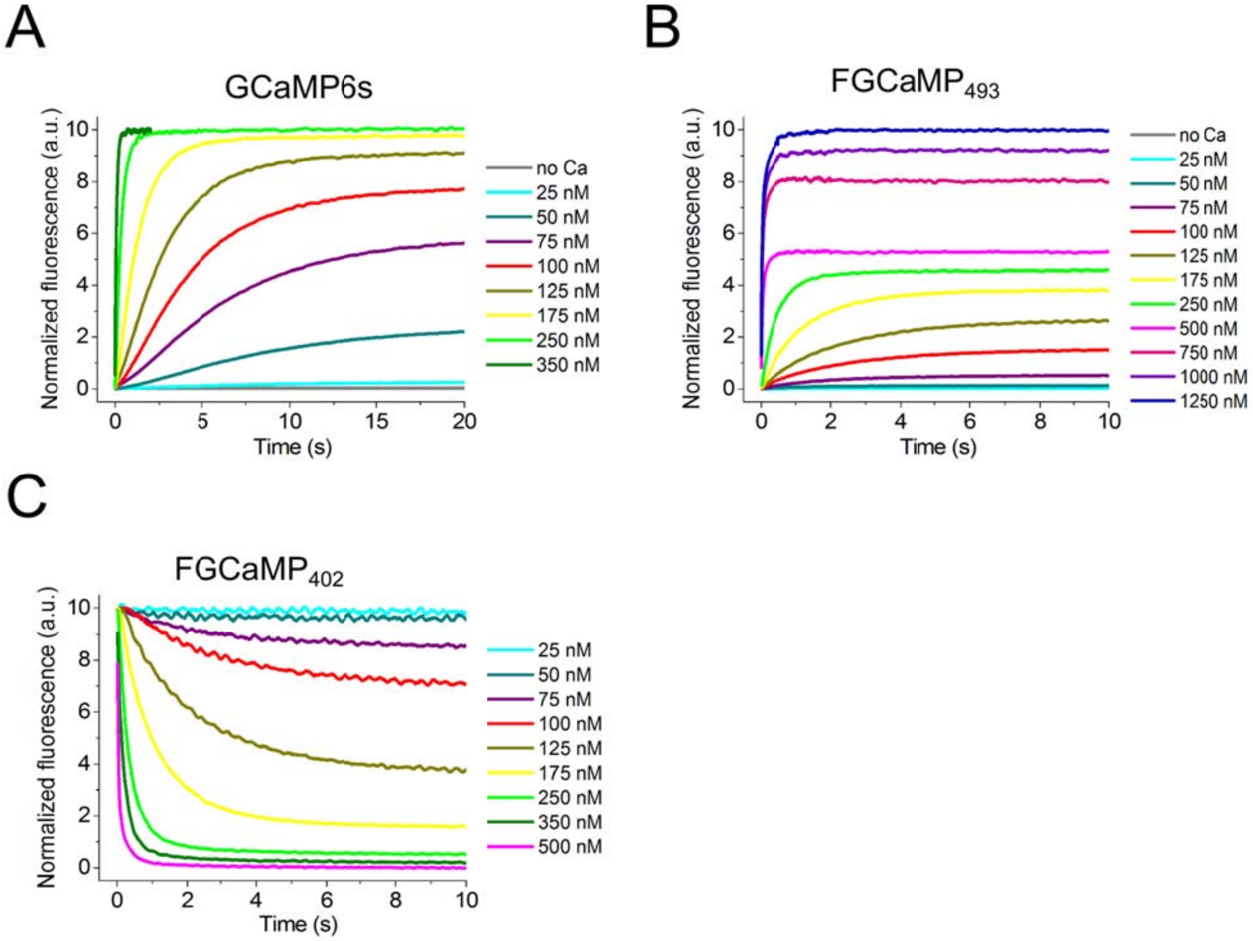

Figure F. Kinetic curves obtained from Ca^{2+}-association experiments. Fluorescence changes are normalized to F_0 of 0 and F_{max} of 10 for GCaMP6s (A) and FGCaMP using excitation at 500 nm (B) and to F_0 of 10 and F_{min} of 0 for FGCaMP using excitation at 402 nm (C). Ca^{2+} concentrations in the mixing chamber are specified in the figure legends.

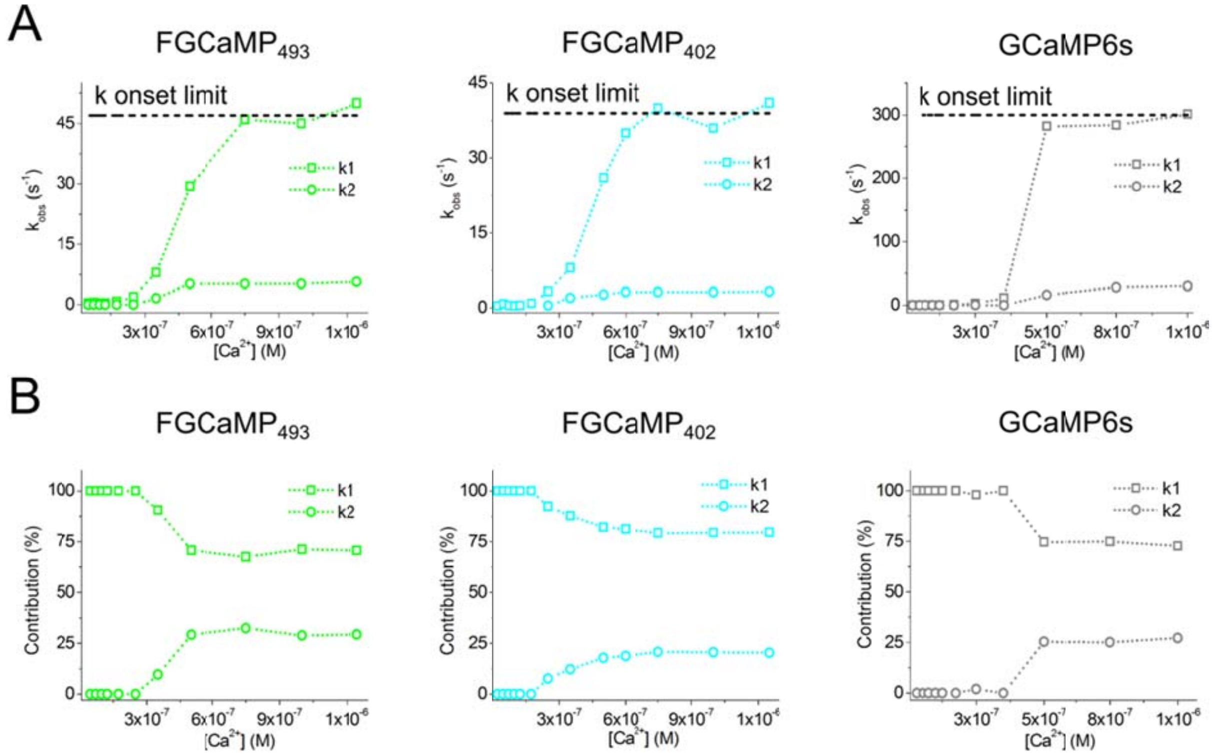

Figure G. Detailed analysis of the onset kinetics for Ca^{2+}-binding. (A) Observed association rate constants (k1, k2) obtained by fitting the kinetic curves to double exponentials. (B) Relative contributions of the k1 and k2 exponentials. Onset rate limit values (k^{onset} limit) correspond to saturation levels of the observed association rate constants (at > 600-800 nM Ca^{2+} concentrations).

A

```
                          10        20
                           |         |
FGCaMP           RRTLHKAIDTVRAINKLREG
GCaMP3           RRKWNKTGHAVRAIGRLSSL
GCaMP6s          RRKWNKTGHAVRAIGRLSSL
```

B

```
                                                           10        20        30
                                                            |         |         |
FGCaMP                                                MADSLTEEQVSEYKEAFSLFDKDGDGQITTKELG
GCaMP3                                                 RDQLTEEQIAEFKEAFSLFDKDGDGTITTKELG
GCaMP6s                                               -PDQLTEEQIAEFKEAFSLFDKDGDGTITTKELG

                       40        50        60        70        80        90
                        |         |         |         |         |         |
FGCaMP           TVMRSLDQNPSESELQDMINEVDADNNGTIDFPEFLTMMARKMKDTDSEEEIREACKVFD
GCaMP6s          TVMRSLGQNPTEAELQDMINEVDADGDGTIDFPEFLTMMARKMKYRDTEEEIREAFGVFD
GCaMP3           TVMRSLGQNPTEAELQDMINEVDADGDGTIDFPEFLTMMARKMKDTDSEEEIREAFRVFD
NTnC                                                             SEEELSECFRTFD

                      100       110       120       130       140
                        |         |         |         |         |
FGCaMP           DRDNNGFISAAELRHVMTSIGEKLTDDEVDEMIREADQDGDGRIDYNEFVQLMMQK
GCaMP6s          DKDGNGYISAAELRHVMTNLGEKLTDEEVDEMIREADIDGDGQVNYEEFVQMMTAK
GCaMP3           DKDGNGYISAAELRHVMTNLGEKLTDEEVDEMIREADIDGDGQVNYEEFVQMMTAK
NTnC             DKDGDGFIDREEFGGIIRLTGEQLTDEDPDEIFGDSDTDKNGRIDFDEFLKMVENVQ
```

Figure H. Alignment of the amino acid sequences of M13-like peptides and CaMs from FGCaMP and the other calcium indicators. (A) Alignment of peptides from CaM-dependent kinase for FGCaMP, GCaMP3, and GCaMP6s indicators. Mutated randomized residues are highlighted with magenta. (B) Alignment of CaMs from FGCaMP, GCaMP3, GCaMP6s, and NTnC. Four EF1-EF4 hands responsible for calcium-binding are highlighted with cyan, mutated residues are highlighted with magenta. Alignment numbering follows that for GCaMP6s.

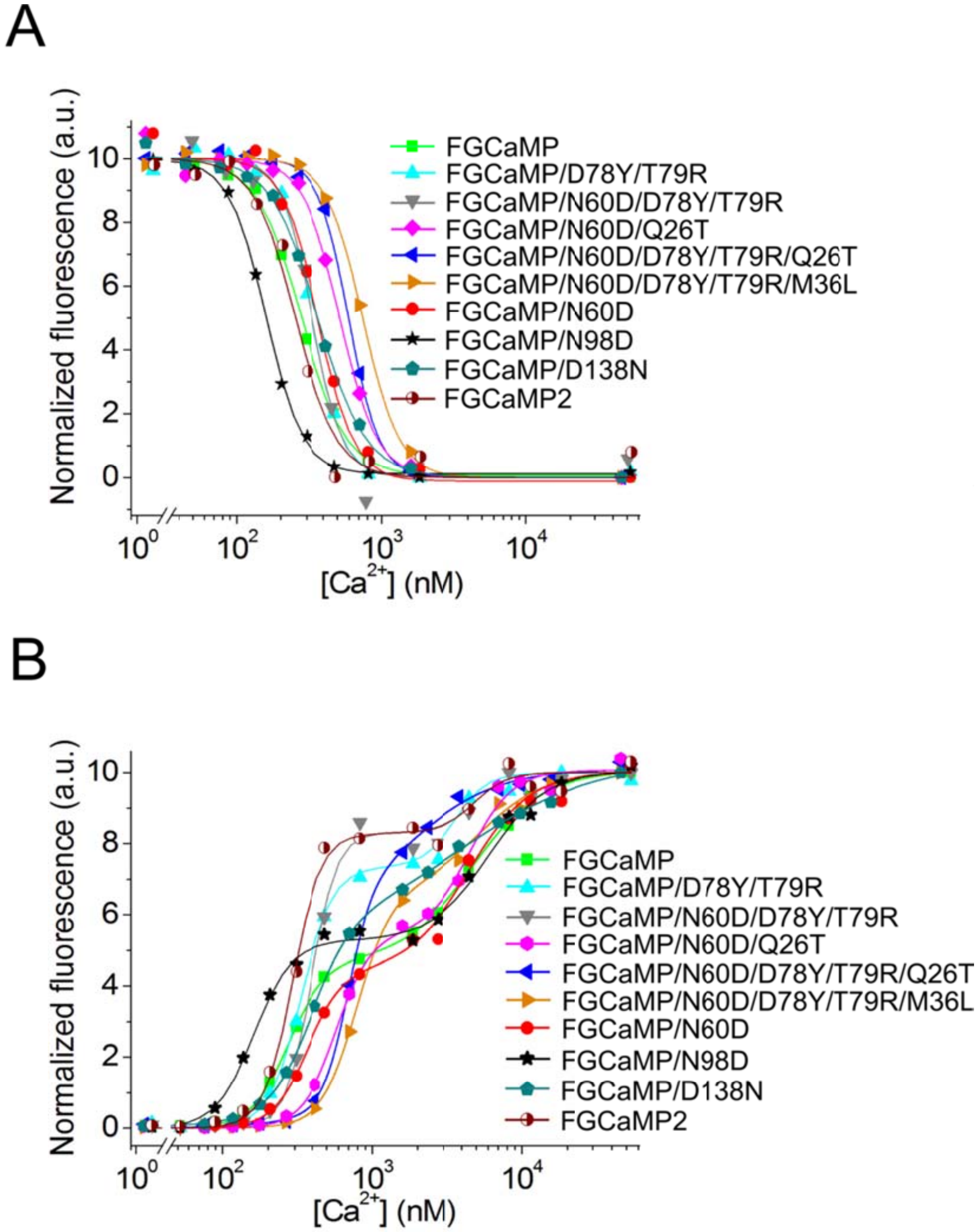

Figure I. Ca^{2+} titration curves for FGCaMP mutants. (A, B) Ca^{2+} titration curves for 402- (A) and 493-forms (B) of FGCaMP mutants using excitation at 402 and 490 nm, respectively. FGCaMP2 corresponds to FGCaMP/N60D/D78Y/T79R/N98D. Alignment numbering follows that for CaM from GCaMP6s. Fluorescence is normalized to maximal and minimal values. SD corresponds to 20%.

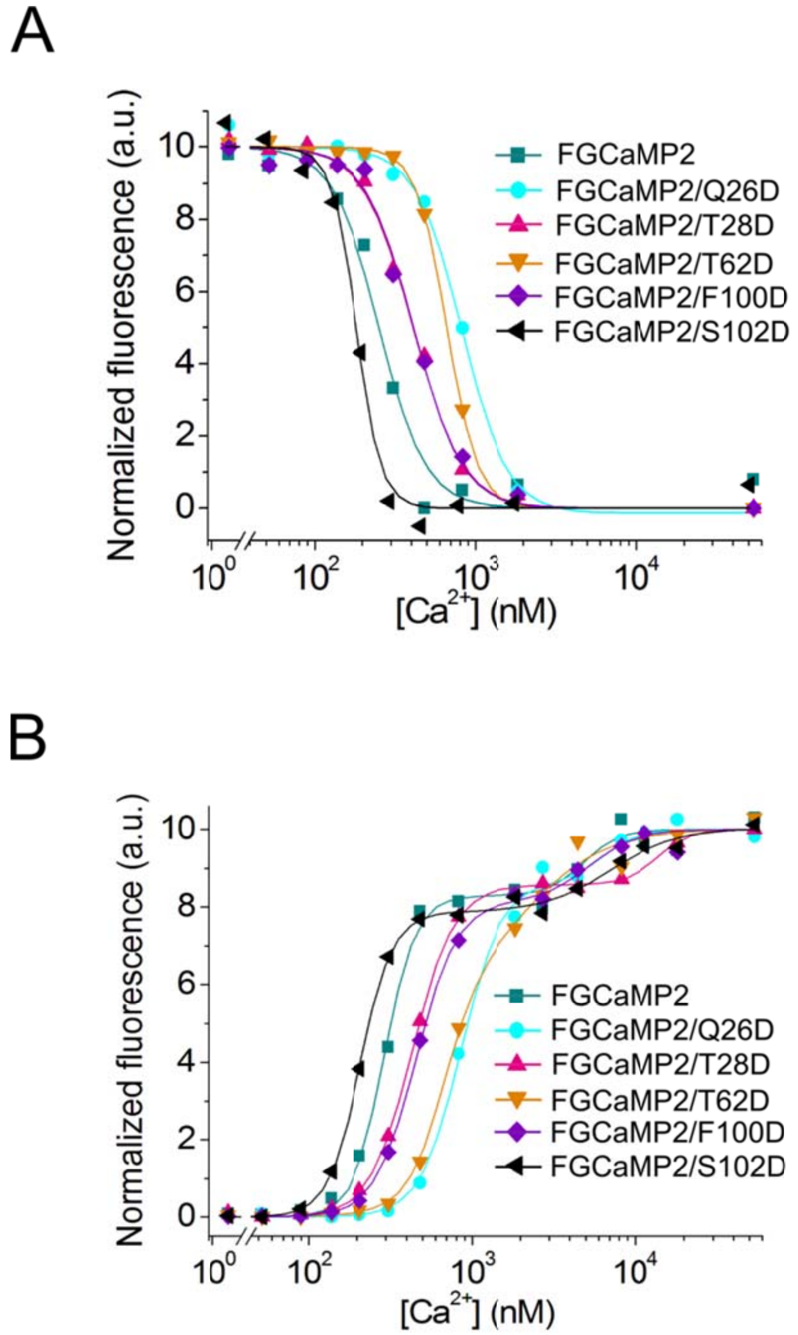

Figure J. Ca^{2+} titration curves for FGCaMP2 mutants with mutations in CaM part. (A, B) Ca^{2+} titration curves for 402- (A) and 493-forms (B) of FGCaMP2 mutants using 402 and 490 nm excitation, respectively. FGCaMP2 corresponds to FGCaMP/N60D/D78Y/T79R/N98D. Alignment numbering follows that for CaM from GCaMP6s. Fluorescence is normalized to maximal and minimal values. SD corresponds to 20%.

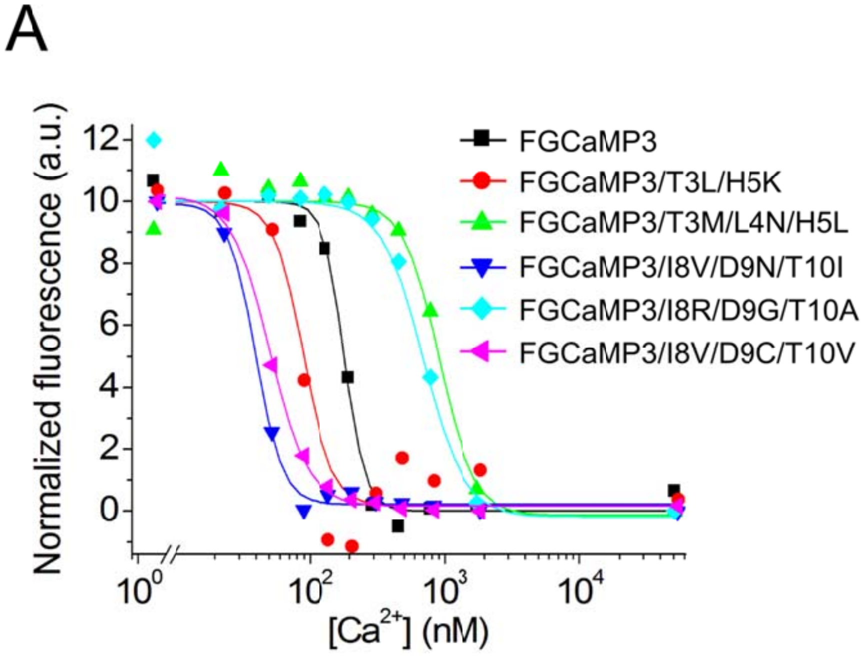

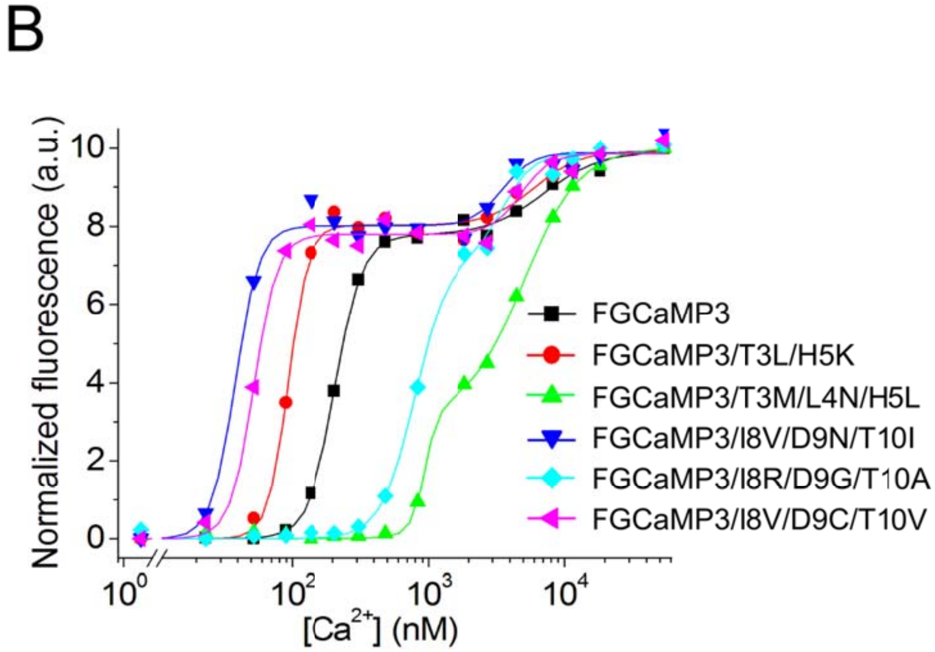

Figure K. Ca^{2+} titration curves for FGCaMP3 variants with mutations in the M13-like peptide. (A, B) Ca^{2+} titration curves for 402- (A) and 493-forms (B) of FGCaMP3 mutants using 402 and 490 nm excitation, respectively. FGCaMP3 corresponds to FGCaMP/N60D/D78Y/T79R/N98D/S102D. Alignment numbering follows that for CaM or M13 peptide from GCaMP6s. Fluorescence is normalized to minimal and maximal fluorescence values. SD corresponds to 20%.

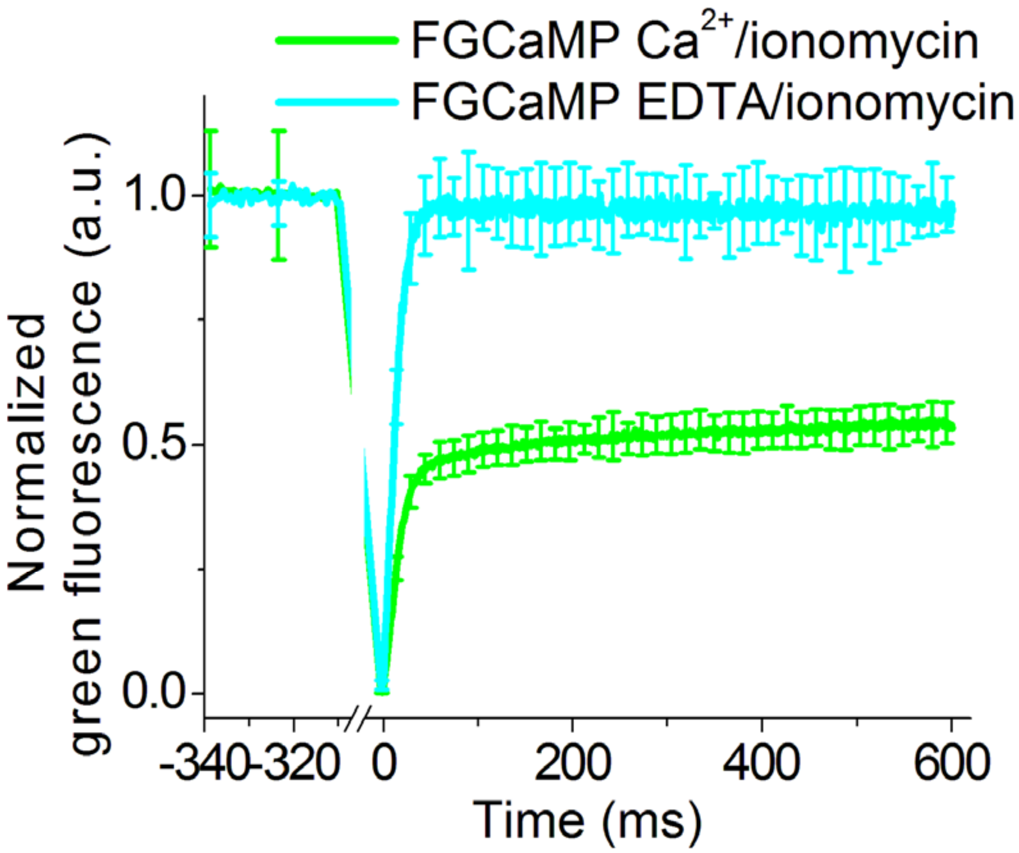

Figure L. Reversible changes in the mobile fraction of the FGCaMP indicator at the transition from high to low Ca^{2+} concentrations in HeLa Kyoto cells studied by FRAP experiments. The graphs illustrate FRAP induced changes in green fluorescence of FGCaMP in response to the addition of 2 mM $CaCl_2$/5 μM ionomycin (high Ca^{2+} concentrations) followed by washing and addition of 1 mM EDTA/5 μM ionomycin (low Ca^{2+} concentrations). Error bars are standard deviations shown for each 20th dot on plots.

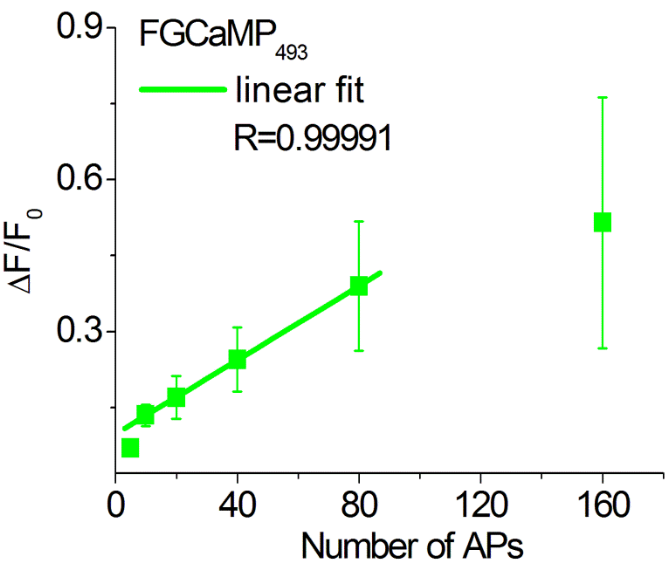

Figure M. Fluorescence changes in response to the external electric field in cultured neurons expressing FGCaMP indicator. Linear region is fitted to a linear equation. APs were induced by electrical pulses of 1 ms, 5 V, and 87 Hz.

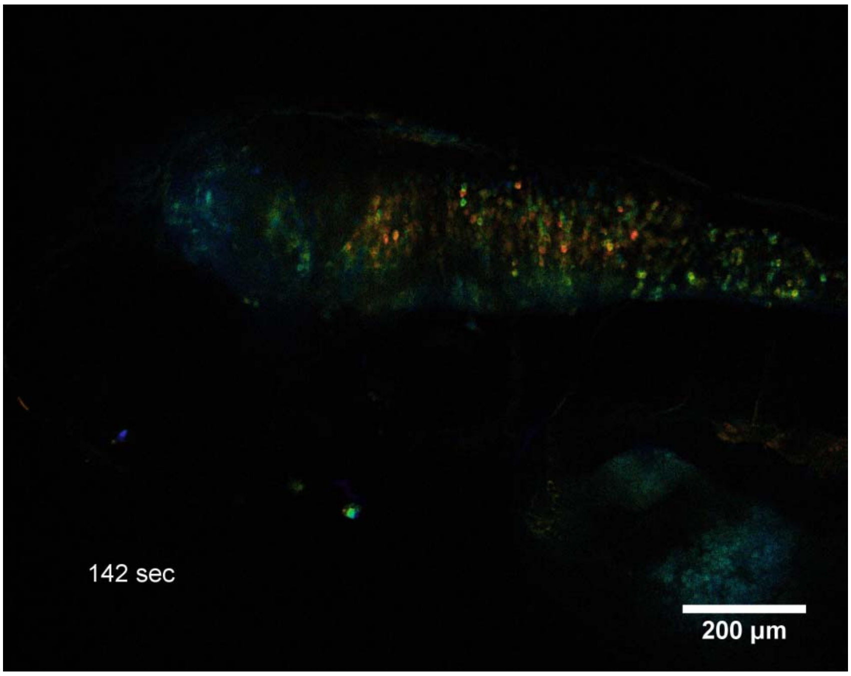

Figure N. Confocal ratiometric calcium imaging with FGCaMP during 4-AP induced neuronal activity in paralyzed larvae embedded in ultra-low-melting agarose gel. Example of frame from S1 Video is shown.

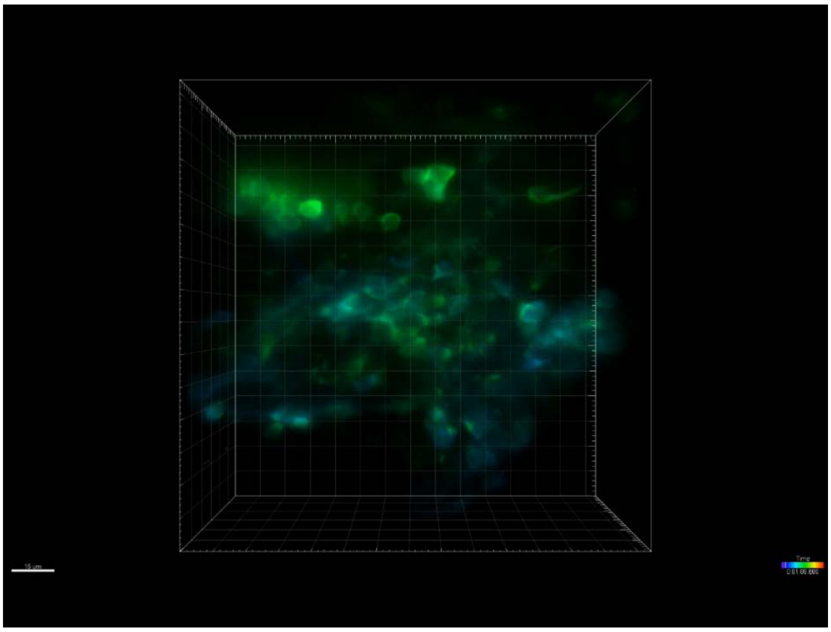

Figure O. Light sheet ratiometric calcium imaging with FGCaMP during 4-AP induced neuronal activity in paralyzed larvae embedded in ultra-low-melting agarose gel. Example of frame from S2 Video is shown.

Table A. Brightness and contrasts for FGCaMP and other ratiometric green and yellow GECIs.

Protein		Brightness	Fluorescence contrast (fold)	Fluorescence Ratio (fold)
**FGCaMP**	**402-form[apo]**	26	6.9±0.5	101
	**493-form[sat]**	49	14.7±0.6	
**GEX-GECO**	**390-form[apo]**	6.7[a]	1.1±0.1	18
	**482-form[sat]**	1[a]	16.1±0.4	
**Ratiometric-pericam [b]**	**418-form [apo]**	7.2	ND	10
	**494-form[sat]**	1.9	ND	
**Y-GECO [c]**	**523-form [apo]**	30	15	200
	**413-form[sat]**	1.2	18	

[a] ref [1]. [b] ref [2]. [c] ref [3]

Table B. K_d values and fluorescence contrasts for 402- and 493-forms of FGCaMP and its mutants.

Protein	402-form		493-form			
	K_{d1} (nM)	Contrast (fold)	K_{d1} (nM)	K_{d2} (μM)	Contrast K_{d1} (fold)	Contrast K_{d2} (fold)
**FGCaMP**	276±9	5.1±0.8	273±7	4.7±0.2	9.3±0.6	1.7±0.1
**FGCaMP/N60D**	370±30	5.5±0.8	370±40	4.4±0.6	9.2±0.6	1.8±0.1
**FGCaMP/N98D**	159±3	5.8±0.9	161±8	6.0±0.5	8.9±0.5	1.7±0.2
**FGCaMP/D138N**	364±13	5.4±0.8	390±9	5.7±2.4	9.9±0.3	1.6±0.1
**FGCaMP/N60D/Q26T**	517±24	3.2±0.5	575±32	4.7±0.2	10.1±0.1	1.8±0.1
**FGCaMP/D78Y/T79R**	330±8	3.7±0.6	333±8	3.6±0.3	10.0±0.4	1.3±0.1
**FGCaMP/N60D/D78Y/T79R**	332±20	3.4±0.5	390±20	4.9±1.1	10.7±0.3	1.2±0.1
**FGCaMP/N60D/D78Y/T79R/Q26T**	600±15	3.4±0.5	693±17	3.2±1.9	11.7±0.3	1.2±0.1
**FGCaMP/N60D/D78Y/T79R/M36L**	743±23	3.4±0.5	788±47	4.6±1.0	13.0±0.6	1.3±0.1
**FGCaMP2** [a]	259±11	3.3±0.5	292±10	4.8±1.1	11.3±0.4	1.2±0.1
**FGCaMP2/Q26D**	824±34	4.3±0.6	857±33	6.4±1.5	10.8±2	1.2±0.1
**FGCaMP2/T28D**	412±13	4.6±0.7	433±4	14±1	13±1	1.1±0.1
**FGCaMP2/T62D**	665±10	3.7±0.6	669±60	2.1±0.8	9.4±1.5	1.4±0.2
**FGCaMP2/F100D**	415±19	3.7±0.6	455±16	5.3±1.1	10.2±0.1	1.2±0.1
**FGCaMP3** [a]	183±8	3.0±0.5	208±4	7±1	10.9±0.2	1.3±0.1
**FGCaMP3/T3L/H5K**	90±20	2.3±0.4	93±2	6±2	6.7±0.3	1.2±0.1
**FGCaMP3/T3M/L4N/H5L**	910±80	3.3±0.5	900±300	5.1±0.3	4.9±0.3	2.2±0.1
**FGCaMP3/I8V/D9N/T10I**	40±2	2.7±0.4	39±3	3.5±0.7	6.3±0.1	1.3±0.1
**FGCaMP3/I8R/D9G/T10A**	700±70	3.0±0.5	790±50	3.6±0.5	4.6±0.3	1.2±0.1
**FGCaMP3/I8V/D9C/T10V**	51±2	4.6±0.7	52±2	4.8±0.7	7.6±0.4	1.2±0.1

[a] FGCaMP2 and FGCaMP3 corresponds to FGCaMP/N60D/D78Y/T79R/N98D and FGCaMP2/S102D, respectively.

Alignment numbering follows that for CaM or M13 peptide from GCaMP6s.

Table C. List of primers.

Primer	Primer sequence (5’-3’)
CaMA-rv	GTATATTTCATTGCTCCCCACCCTTTCCGC
CaMN-BglII	CGAGATCTATGGCCGACTCTCTGACCGAAGAGCAAG
CaMN-HindIII reverse	CCAAGCTTTTATTTTTGCATCATGAGCTGGACGAACTCGTTG
CaMK-BglII	CGAGATCTATGTCCGATAAATTGTCCGAAGCCCAAATTTC
CaMK-HindIII reverse	CCAAGCTTCTATTTATTAACTAGGAGATTGGAAAATTCCTTG
M13Fc-BglII forward	CAGAGATCTAAAAGACGGGCGATCAAGAACAAG
M13Fc reverse	CCGCAACACCTGGAAGACACGCGAGAGACGTCCGATGGCAAGAATCTTGTTC TTGATCGCCCG
M13Fc-cpEGFP forward	GTCTTCCAGGTGTTGCGGNNSNNSAACGTCTATATCAAGGCC
M13Fc-cpEGFP reverse	GGCCTTGATATAGACGTTSNNSNNCCGCAACACCTGGAAGAC
cpEGFP-CaMF forward	CACAAGCTGGAGTACAACNNSNNSNNSATGGCCGACTCTCTGACCGAAGAGC AAG
cpEGFP-CaMF reverse	CTTGCTCTTCGGTCAGAGAGTCGGCCATSNNSNNSNNGTTGTACTCCAGCTTG TG
M13Fk-BglII forward	CAGAGATCTCGTCGAACGCTGCACAAAGCC
M13Fk reverse	CCCTCGCGGAGCTTGTTGATGGCGCGGACGGTATCGATGGCTTTGTGCAGCG TTCG
M13Fk-cpEGFP forward	CAACAAGCTCCGCGAGGGANNSNNSAACGTCTATATCAAGGCC
M13Fk-cpEGFP reverse	GGCCTTGATATAGACGTTSNNSNNTCCCTCGCGGAGCTTGTTG
M13KPc-BglII forward	CAGAGATCTAAGAAAGTGGTACTCAGAAATAAG
M13KPc reverse	GGAGAACTTGATACATCCTGGATACTCTTCCAATTGCCAGAATCTTATTTCTGA GTACCACTTTC
M13KPc-cpEGFP forward	GGATGTATCAAGTTCTCCGTNNSNNSAACGTCTATATCAAGGCC
M13KPc-cpEGFP reverse	GGCCTTGATATAGACGTTSNNSNNACGGAGAACTTGATACATCC
cpEGFP-CaMKP forward	CACAAGCTGGAGTACAACNNSNNSNNSATGTCCGATAAATTGTCCGAAGCCCA AATTTC
cpEGFP-CaMKP reverse	GAAATTTGGGCTTCGGACAATTTATCGGACATSNNSNNSNNGTTGTACTCCAG CTTGTG
M13KPk-BglII forward	CAGAGATCTAGATCCAAGTTCAAGCAAG
M13KPk reverse	GTTCTTCAATCTGTTTTGCATCTTGACAATCTCGATGACTTGCTTGAACTTGGA TC
M13KPk-cpEGFP forward	GCAAAACAGATTGAAGAACCTCNNSNNSAACGTCTATATCAAGGCC
M13KPk-cpEGFP reverse	GGCCTTGATATAGACGTTSNNSNNGAGGTTCTTCAATCTGTTTTGC
FCaMP-M36L forward	CAAGGAGCTCGGCACTGTGCTGCGCTCCCTTGACCAGAAC
FCaMP-M36L reverse	GTTCTGGTCAAGGGAGCGCAGCACAGTGCCGAGCTCCTTG
FCaMP-N60D forward	GAGGTTGACGCTGACAACGACGGAACGATCGACTTCC
FCaMP-N60D	GGAAGTCGATCGTTCCGTCGTTGTCAGCGTCAACCTC

reverse	
FCaMP-78YR forward	GATGGCTCGTAAGATGAAGTACAGAGACACCGAGGAGGAAATCCGCG
FCaMP-78YR reverse	CGCGGATTTCCTCCTCGGTGTCTCTGTACTTCATCTTACGAGCCATC
FCaMP-Q26T forward	CAAGGATGGCGATGGCACGATCACCACCAAGGAGCTC
FCaMP-Q26T reverse	GAGCTCCTTGGTGGTGATCGTGCCATCGCCATCCTTG
FCaMP-N98D forward	GTCTTCGACCGCGACAACGATGGTTTTATCTCCGCCGCG
FCaMP-N98D reverse	CGCGGCGGAGATAAAACCATCGTTGTCGCGGTCGAAGAC
FCaMP-D138N reverse	GAGCTGGACGAACTCGTTGTAGTTGATGCGGCCATCACCGTC
FC-102D forward	GACAACGATGGTTTTATCGACGCCGCGGAGCTGCGCCAC
FC-102D reverse	GTGGCGCAGCTCCGCGGCGTCGATAAAACCATCGTTGTC
FC-BglII-M13NNS1 forward	GGCAGATCTATGCGTAGCCGTCGANNSNNSNNSAAAGCCATCGATACCGTC
FC-BglII-M13NNS2 forward	GGCAGATCTATGCGTAGCCGTCGAACGCTGCACAAAGCCNNSNNSNNSGTCCGCGCCATCAACAAG
G-FCaMP-BamHI-NheI forward	TGGGGATCCGGCTAGGCCACCATGCGTCGAACGCTGCACAAAGCC
GEX-GECO-BglII forward	CAGAGATCTATGGTCGACTCATCACGTC
GEX-GECO-EcoRI reverse	CAGGAATTCTCACTTCGCTGTCATCATTTG

References

1. Zhao Y, Araki S, Wu J, Teramoto T, Chang YF, Nakano M, et al. (2011) An expanded palette of genetically encoded Ca(2) indicators. Science 333: 1888-1891.
2. Nagai T, Sawano A, Park ES, Miyawaki A (2001) Circularly permuted green fluorescent proteins engineered to sense Ca2+. Proceedings of the National Academy of Sciences of the United States of America 98: 3197-3202.
3. Zhao Y, Abdelfattah AS, Ruangkittisakul A, Ballanyi K, Campbell RE, Harrison DJ (2014) Microfluidic cell sorter-aided directed evolution of a protein-based calcium ion indicator with an inverted fluorescent response. Integrative biology : quantitative biosciences from nano to macro 6: 714-725.
